# Supplementary material for: A novel somatosensory spatial navigation system outside the hippocampal formation
Source: Cell Res. 2021 Jan 18;31(6):649–63. doi: 10.1038/s41422-020-00448-8 (PMC8169756; doi:10.1038/s41422-020-00448-8)
Supplement: Supplementary file 1 — Figure S1 [file 41422_2020_448_MOESM1_ESM.pdf]

Supplementary information, Fig. S1

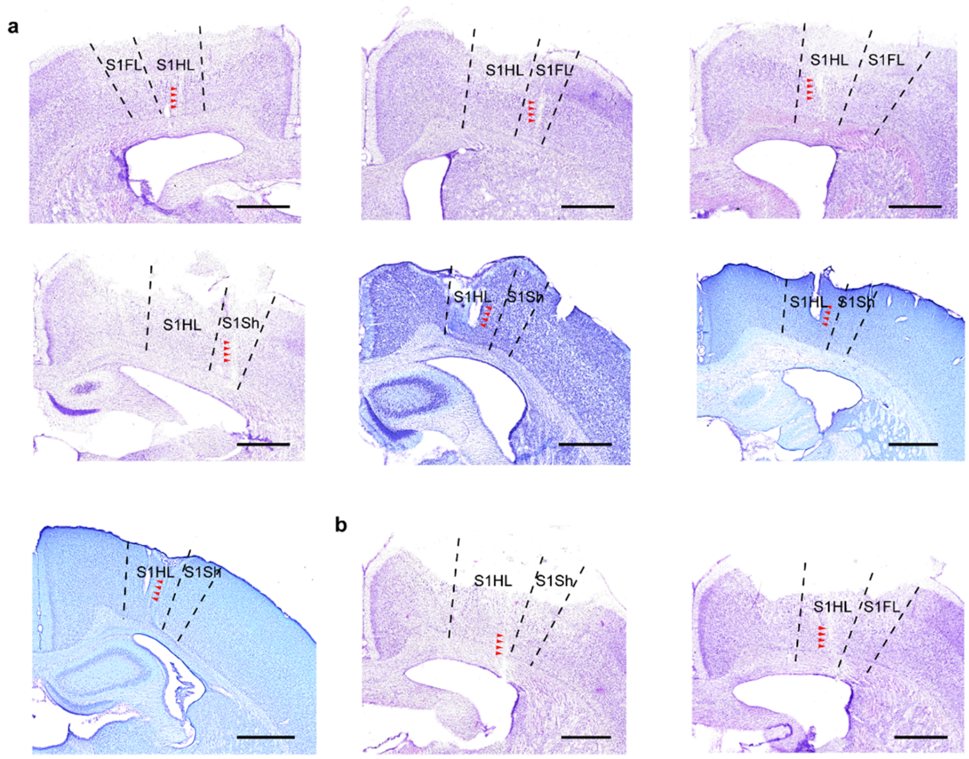

Supplementary information, Fig. S1. Electrode track and recording locations in the primary somatosensory cortex.

a Cresyl violet-stained coronal brain sections show recording electrode tracks (arrowheads) and final recording positions in seven rats with tetrodes implanted in the rat primary somatosensory cortex.

b Cresyl violet-stained coronal brain sections showing representative recording locations (arrowheads) for two additionally implanted rats in the whisker trimming experiment. Dashed lines depict the boundaries of the hindlimb region (S1HL), shoulder region (S1Sh) and forelimb (S1FL) region of the primary somatosensory cortex according to the rat brain atlas of Paxinos and Watson (2007). Scale bar, 1 mm.
