# Supplementary material for: A novel somatosensory spatial navigation system outside the hippocampal formation
Source: Cell Res. 2021 Jan 18;31(6):649–63. doi: 10.1038/s41422-020-00448-8 (PMC8169756; doi:10.1038/s41422-020-00448-8)
Supplement: Supplementary file 2 — Figure S2 [file 41422_2020_448_MOESM2_ESM.pdf]

Supplementary information, Fig. S2

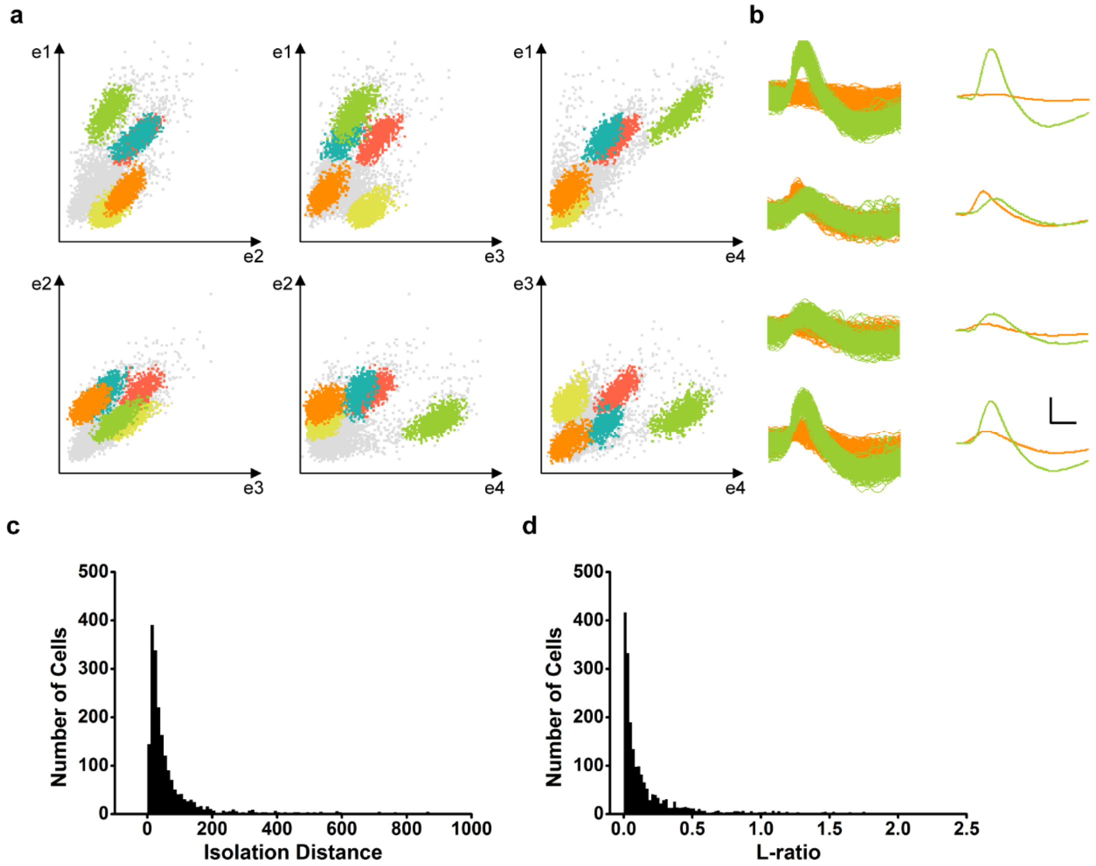

Supplementary information, Fig. S2. Cluster diagrams, waveforms and isolation quality of spike clusters recorded from the somatosensory cortex.

a Scatterplots show the relationship between peak-to-trough amplitudes for all spikes for each wire (e1-e4) in a tetrode.

b Overlaid and mean waveforms from two separated green and orange clusters in the scatterplots are shown for four electrodes from the same tetrode. Scale bar, 150 μV, 200 μs.

c The distribution of isolation distance for identified somatosensory units.

d Same as (**c**) for the L-ratio.
