# Supplementary material for: A novel somatosensory spatial navigation system outside the hippocampal formation
Source: Cell Res. 2021 Jan 18;31(6):649–63. doi: 10.1038/s41422-020-00448-8 (PMC8169756; doi:10.1038/s41422-020-00448-8)
Supplement: Supplementary file 3 — Figure S3 [file 41422_2020_448_MOESM3_ESM.pdf]

Supplementary information, Fig. S3

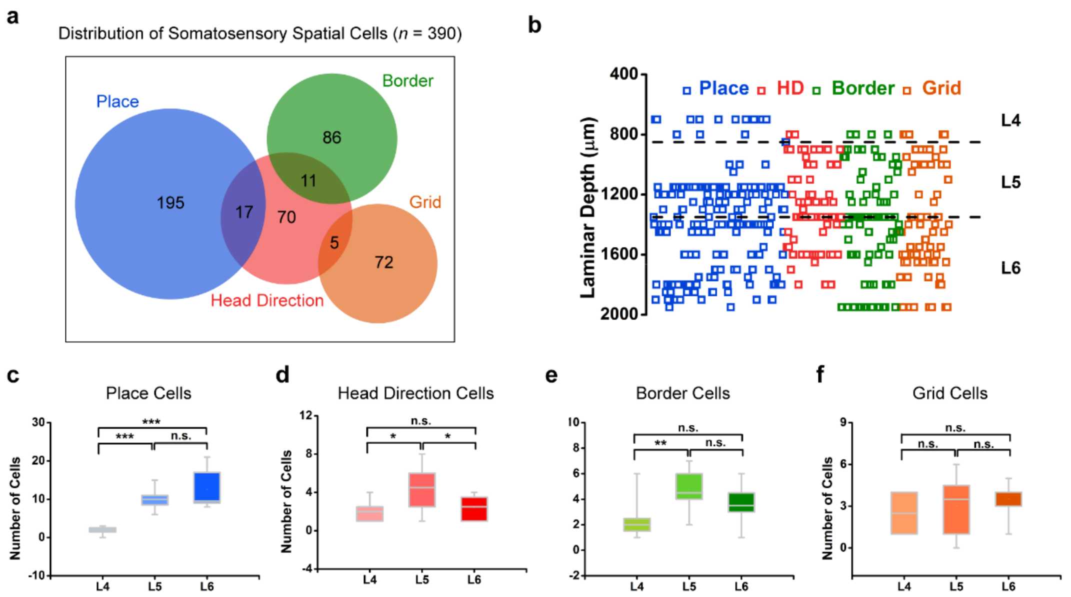

Supplementary information, Fig. S3. Layer distribution of four different somatosensory spatial cell types.

a Venn diagram displaying the distribution of four functionally distinct somatosensory spatial cell types.

b The approximate distributed laminar locations of all recorded functionally distinct somatosensory spatial cells.

c-f The layer distribution of average number of identified somatosensory place cells (**c**), head direction cells (**d**), border cells (**e**) and grid cells (**f**) across different implanted rats.
