# Supplementary material for: A novel somatosensory spatial navigation system outside the hippocampal formation
Source: Cell Res. 2021 Jan 18;31(6):649–63. doi: 10.1038/s41422-020-00448-8 (PMC8169756; doi:10.1038/s41422-020-00448-8)
Supplement: Supplementary file 4 — Figure S4 [file 41422_2020_448_MOESM4_ESM.pdf]

Supplementary information, Fig. S4

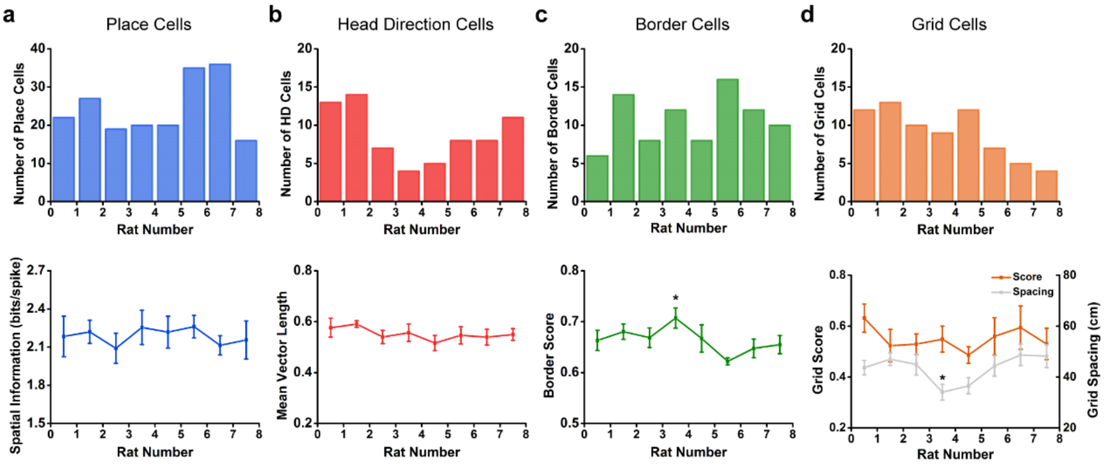

Supplementary information, Fig. S4. Distribution of number and characteristics of four different somatosensory spatial cell types.

a-d Distribution of number (upper panels) and spatial characteristics (bottom panels) of identified somatosensory place cells (**a**), head direction cells (**b**), border cells (**c**) and grid cells (**d**) across different animals.
