# Supplementary material for: A novel somatosensory spatial navigation system outside the hippocampal formation
Source: Cell Res. 2021 Jan 18;31(6):649–63. doi: 10.1038/s41422-020-00448-8 (PMC8169756; doi:10.1038/s41422-020-00448-8)
Supplement: Supplementary file 5 — Figure S5 [file 41422_2020_448_MOESM5_ESM.pdf]

**Supplementary information, Fig. S5**

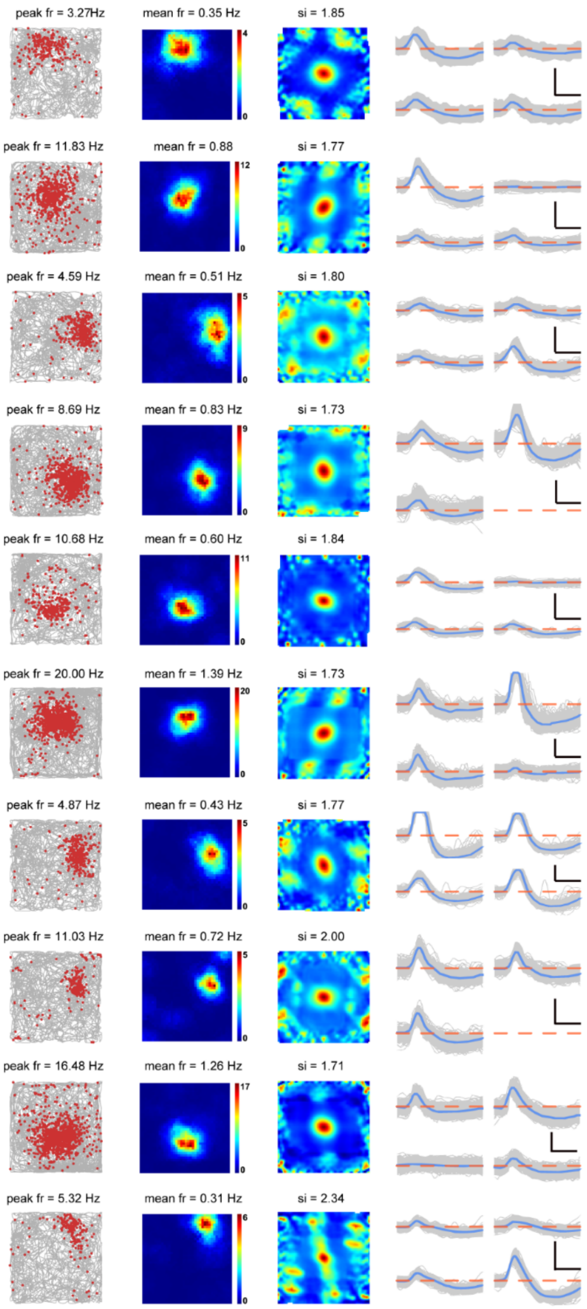


**Supplementary information, Fig. S5. More examples of identified place cells recorded from the somatosensory cortex.**

**a-c** Representative somatosensory place cells with bin coverage over 90%. Trajectory (grey line) with superimposed spike locations (red dots) (left column); heat maps of spatial firing rate (middle column) and autocorrelation (right column) are color-coded with dark blue indicating minimal firing rate and dark red indicating maximal firing rate. The scale of the autocorrelation maps is twice that of the spatial firing rate maps.

Peak firing rate (fr), mean firing rate (fr) and spatial information (si) for each representative cell are labelled at the top of the panels. Spike waveforms on four electrodes are shown on the right column. The zero microvolt horizontal baseline is drawn with the orange dashed lines for the spike waveforms on all four electrodes. Scale bar, 150 μV, 300 μs.
