# Supplementary material for: A novel somatosensory spatial navigation system outside the hippocampal formation
Source: Cell Res. 2021 Jan 18;31(6):649–63. doi: 10.1038/s41422-020-00448-8 (PMC8169756; doi:10.1038/s41422-020-00448-8)
Supplement: Supplementary file 6 — Figure S6 [file 41422_2020_448_MOESM6_ESM.pdf]

Supplementary information, Fig. S6

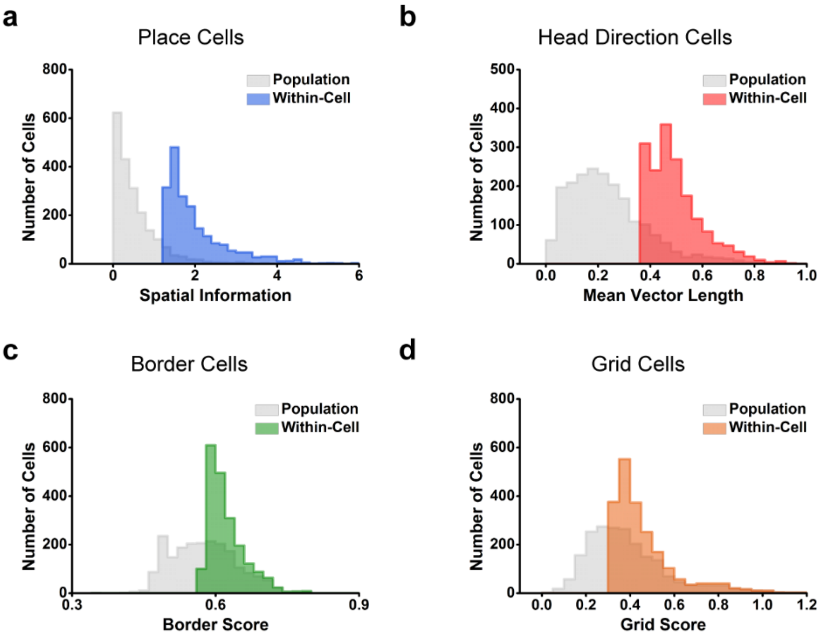

Supplementary information, Fig. S6. Comparison of spatial threshold defined by population shuffling and within-cell shuffling for four different somatosensory spatial cell types.

a-d Histograms showing the 99th percentile significance level of each randomly shuffled distribution for population shuffling and within-cell shuffling of all 2025 identified single units for identified somatosensory place cells (**a**), head direction cells (**b**), border cells (**c**) and grid cells (**d**).
