# Supplementary material for: A novel somatosensory spatial navigation system outside the hippocampal formation
Source: Cell Res. 2021 Jan 18;31(6):649–63. doi: 10.1038/s41422-020-00448-8 (PMC8169756; doi:10.1038/s41422-020-00448-8)
Supplement: Supplementary file 7 — Figure S7 [file 41422_2020_448_MOESM7_ESM.pdf]

Supplementary information, Fig. S7

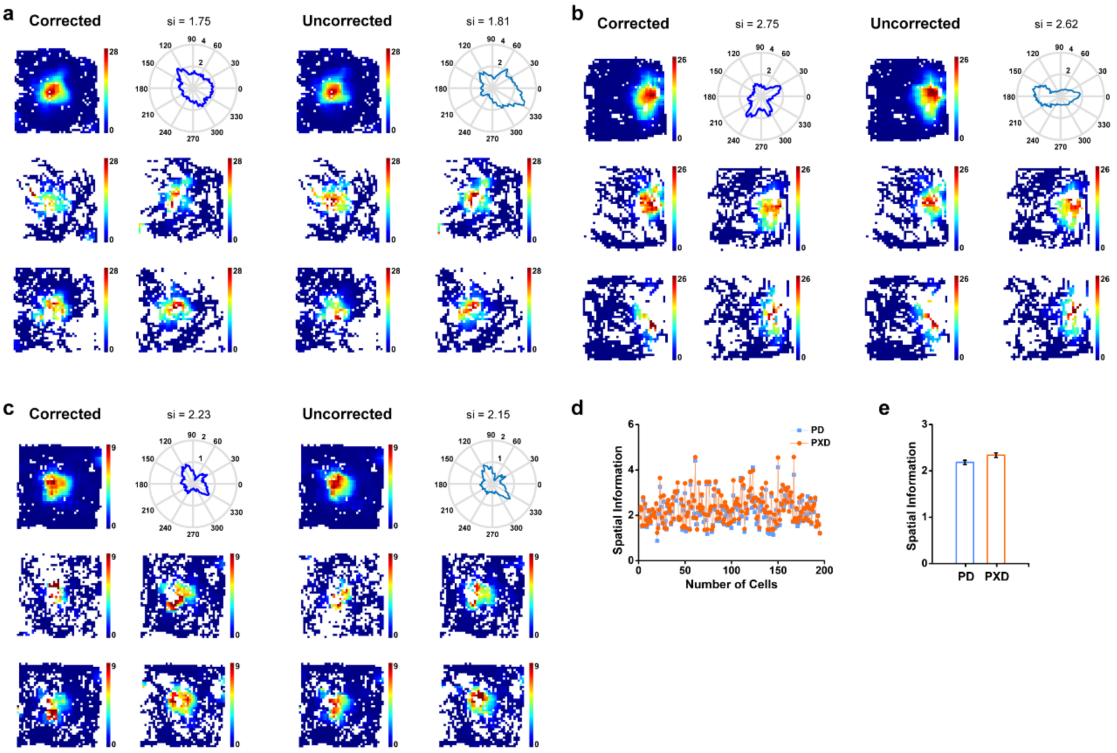

Supplementary information, Fig. S7. Quantification of spatial responses of somatosensory place cells using the maximum likelihood factorial model.

a-c Firing rate maps of three representative place cells from Fig. 1b. Left column shows the corrected locational and directional firing rate maps using the maximum-likelihood correction approach; right column shows the corresponding uncorrected firing rate maps. Spatial firing rate maps in four directions are shown in the lower panels. Note the similar locational responses in four different directions.

d, e The distribution and the average value of the spatial information of recorded S1 place cells before (PD) and after (PXD) applying the maximum-likelihood correction algorithm.
