# Supplementary material for: A novel somatosensory spatial navigation system outside the hippocampal formation
Source: Cell Res. 2021 Jan 18;31(6):649–63. doi: 10.1038/s41422-020-00448-8 (PMC8169756; doi:10.1038/s41422-020-00448-8)
Supplement: Supplementary file 8 — Figure S8 [file 41422_2020_448_MOESM8_ESM.pdf]

Supplementary information, Fig. S8

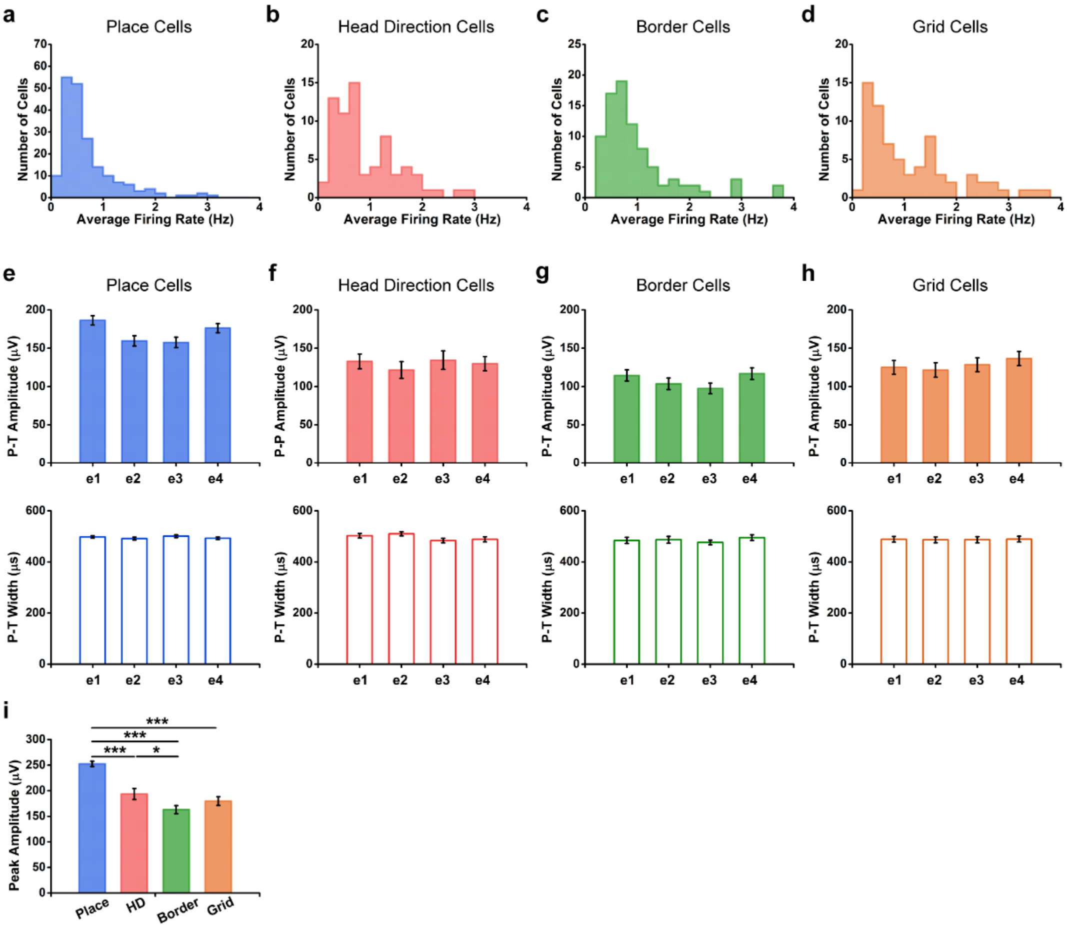

Supplementary information, Fig. S8. Distribution of the average firing rate and summary of spike waveform for four different somatosensory spatial cell types.

a-d Histograms showing the average firing rate of identified somatosensory place cells (**a**), head direction cells (**b**), border cells (**c**) and grid cells (**d**).

e-h Histograms showing the peak-to-peak amplitudes (upper panels) and peak to trough width (bottom panels) of spike waveforms on four electrodes of identified somatosensory place cells (**e**), head direction cells (**f**), border cells (**g**) and grid cells (**h**).

i The comparison of the highest peak amplitude on four electrodes for four different somatosensory spatial cell types. *$P < 0.05$, ***$P < 0.001$, two-tailed unpaired *t*-test.
