# Supplementary material for: A novel somatosensory spatial navigation system outside the hippocampal formation
Source: Cell Res. 2021 Jan 18;31(6):649–63. doi: 10.1038/s41422-020-00448-8 (PMC8169756; doi:10.1038/s41422-020-00448-8)
Supplement: Supplementary file 9 — Figure S9 [file 41422_2020_448_MOESM9_ESM.pdf]

Supplementary information, Fig. S9

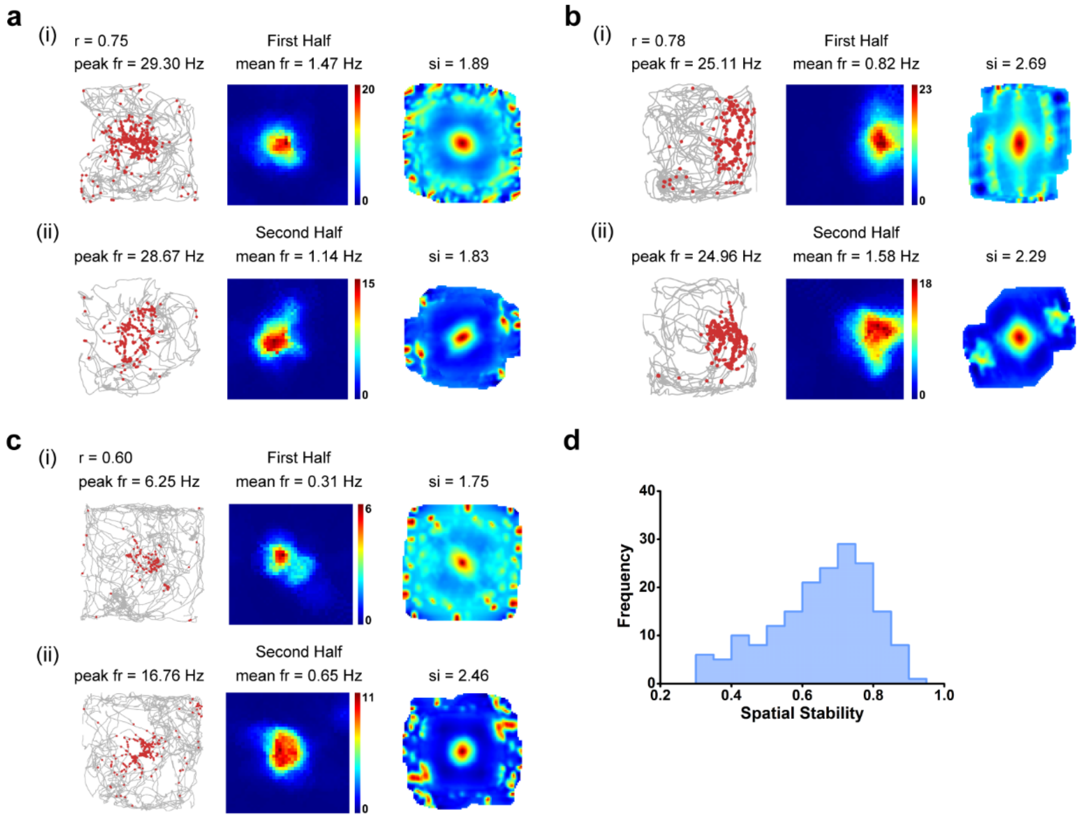

Supplementary information, Fig. S9. Spatial stability of somatosensory place cells.

a-c Intra-trial spatial stability between the first and second halves of three representative place cells from Fig. 1b. Trajectory (grey line) with superimposed spike locations (red dots) (left column); rate maps (middle column) and autocorrelation maps (right column) for the first half (**i**) and the second half (**ii**) of the trials. Firing rate is color-coded with blue indicating minimum firing rate and red indicating maximum firing rate. The scale of the autocorrelation maps is twice that of the spatial firing rate maps. Peak firing rate (fr), mean firing rate (fr) and spatial information (si) are labelled at the top of the plots. Pearson's correlation coefficients of firing rate maps between the first and second halves are indicated with *r* at the top-left corner.

d Distribution of spatial stability of firing rate maps between the first and second halves of all identified somatosensory place cells.
