# Supplementary material for: A novel somatosensory spatial navigation system outside the hippocampal formation
Source: Cell Res. 2021 Jan 18;31(6):649–63. doi: 10.1038/s41422-020-00448-8 (PMC8169756; doi:10.1038/s41422-020-00448-8)
Supplement: Supplementary file 10 — Figure S10 [file 41422_2020_448_MOESM10_ESM.pdf]

Supplementary information, Fig. S10

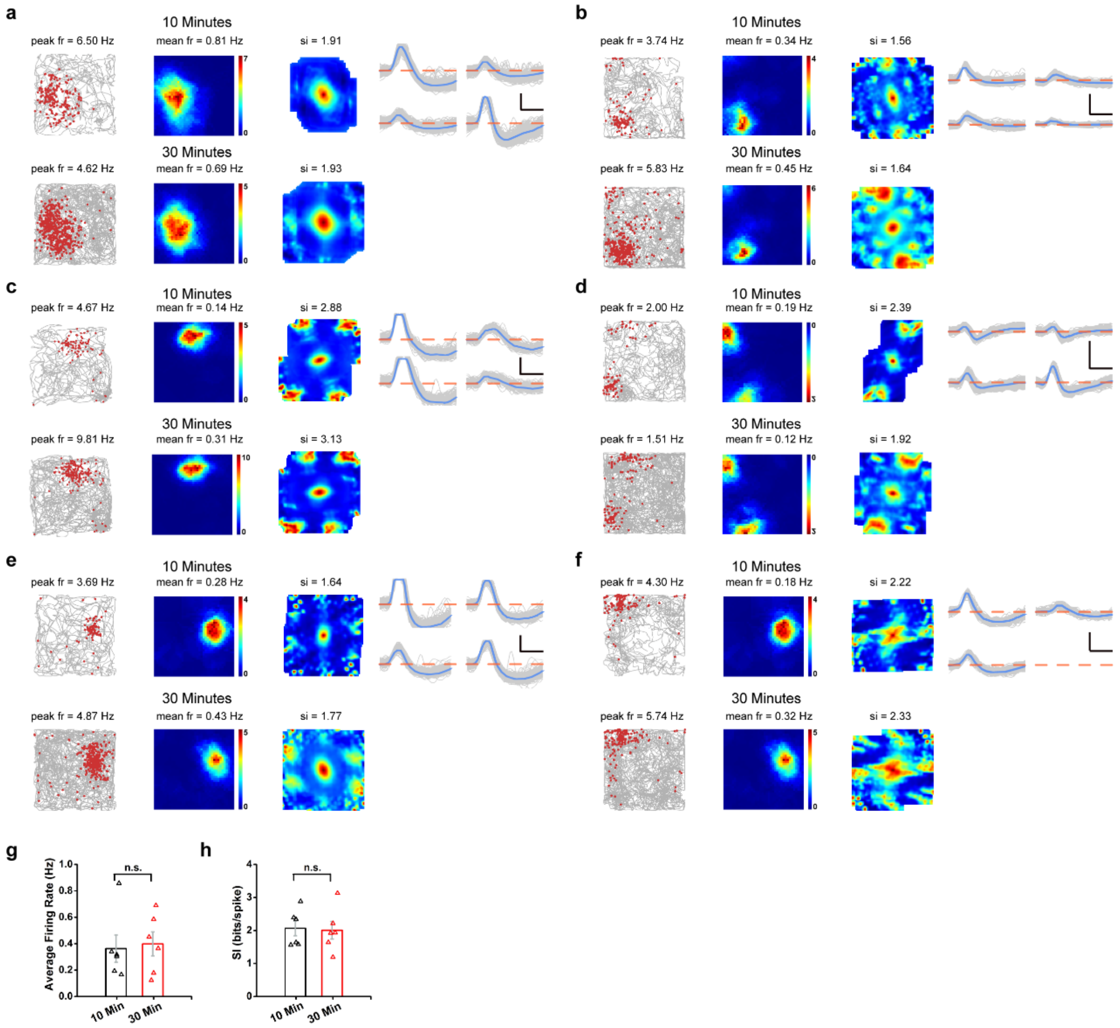

Supplementary information, Fig. S10. Persistence of somatosensory place fields from longer recording sessions.

a-f Spatial stability of three representative somatosensory place cells between the short (top panels) and longer (bottom panels) recording sessions. Trajectory (grey line) with superimposed spike locations (red dots) (left column); spatial firing rate maps (middle column) and autocorrelation diagrams (right column). Firing rate is color-coded with blue indicating minimum firing rate and red indicating maximum firing rate. The scale of the autocorrelation maps is twice that of the spatial firing rate maps. Peak firing rate (fr), mean firing rate (fr) and spatial information (si) for each recording session are labelled at the top of the panels. Spike waveforms on four electrodes are shown on the right column. The zero microvolt horizontal baseline is drawn with the orange dashed lines for the spike waveforms on all four electrodes. Scale bar, 150 µV, 300 µs.

g, **h** The comparison of the average firing rate and the average spatial information during 10 min recordings versus 30 min extended longer recordings.
