# Supplementary material for: A novel somatosensory spatial navigation system outside the hippocampal formation
Source: Cell Res. 2021 Jan 18;31(6):649–63. doi: 10.1038/s41422-020-00448-8 (PMC8169756; doi:10.1038/s41422-020-00448-8)
Supplement: Supplementary file 11 — Figure S11 [file 41422_2020_448_MOESM11_ESM.pdf]

Supplementary information, Fig. S11

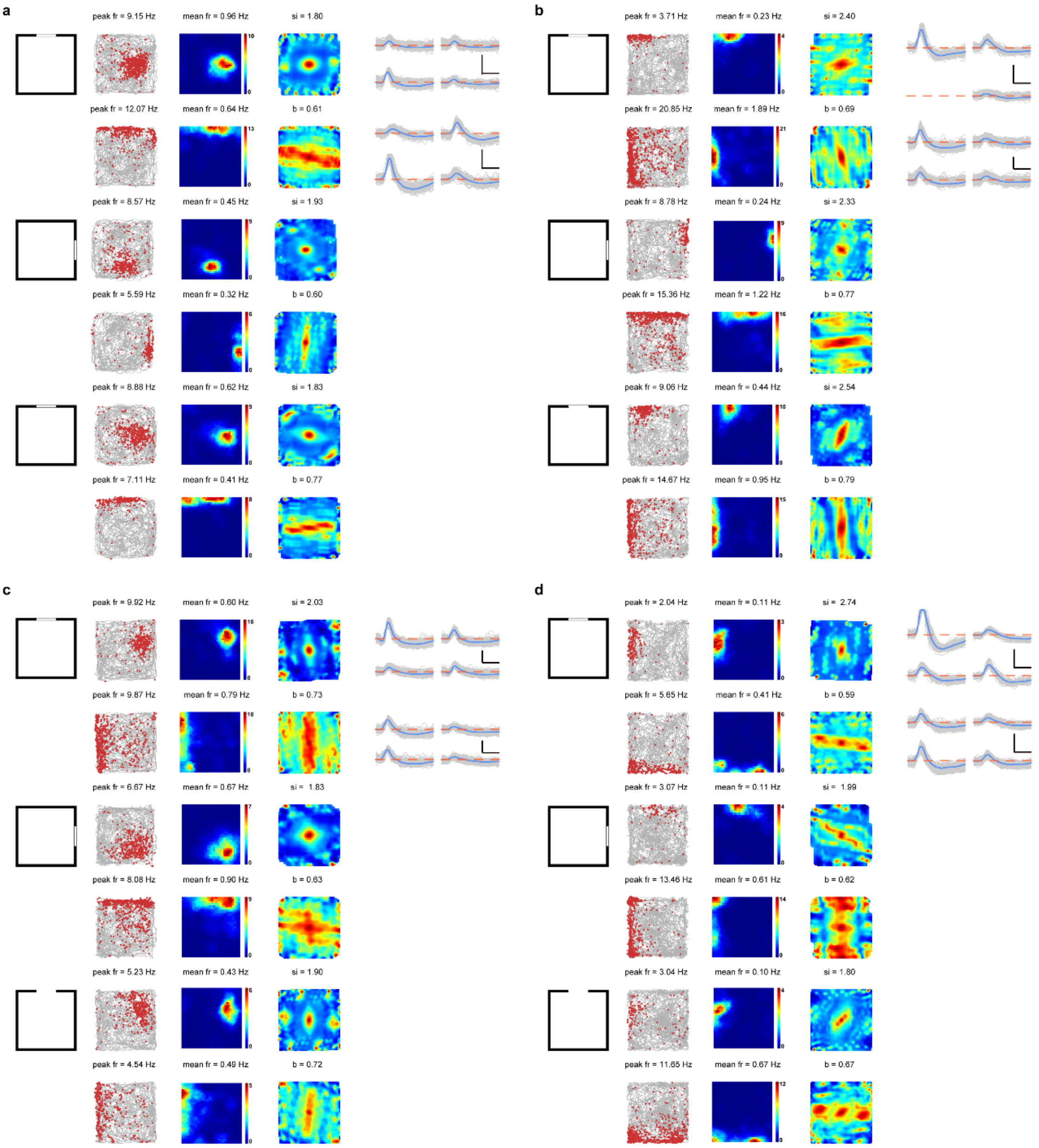

Supplementary information, Fig. S11. Cue-rotation manipulations of simultaneously recorded somatosensory place cell and border cell.

a-d Each panel shows the response of the same somatosensory place cell and co-recorded border cell during three sessions of the cue-rotation condition. Top panels, before the cue-rotation; middle panels, counterclockwise or clockwise 90° of cue-rotation; bottom panels, cue-rotation back to the original condition. The cue card is represented by a white arc in each panel. The experimental diagram (left column); trajectory (grey line) with superimposed spike locations (red dots) (middle left column); spatial firing rate maps (middle right column) and autocorrelation diagrams (right

column). Firing rate is color-coded with blue indicating minimum firing rate and red indicating maximum firing rate. The scale of the autocorrelation maps is twice that of the spatial firing rate maps. Peak firing rate (fr), mean firing rate (fr) and spatial information (si) for each recording session are labelled at the top of the panels. Spike waveforms on four electrodes are shown on the right column. The zero microvolt horizontal baseline is drawn with the orange dashed lines for the spike waveforms on all four electrodes. Scale bar, 150 μV, 300 μs.
