# Supplementary material for: A novel somatosensory spatial navigation system outside the hippocampal formation
Source: Cell Res. 2021 Jan 18;31(6):649–63. doi: 10.1038/s41422-020-00448-8 (PMC8169756; doi:10.1038/s41422-020-00448-8)
Supplement: Supplementary file 12 — Figure S12 [file 41422_2020_448_MOESM12_ESM.pdf]

Supplementary information, Fig. S12

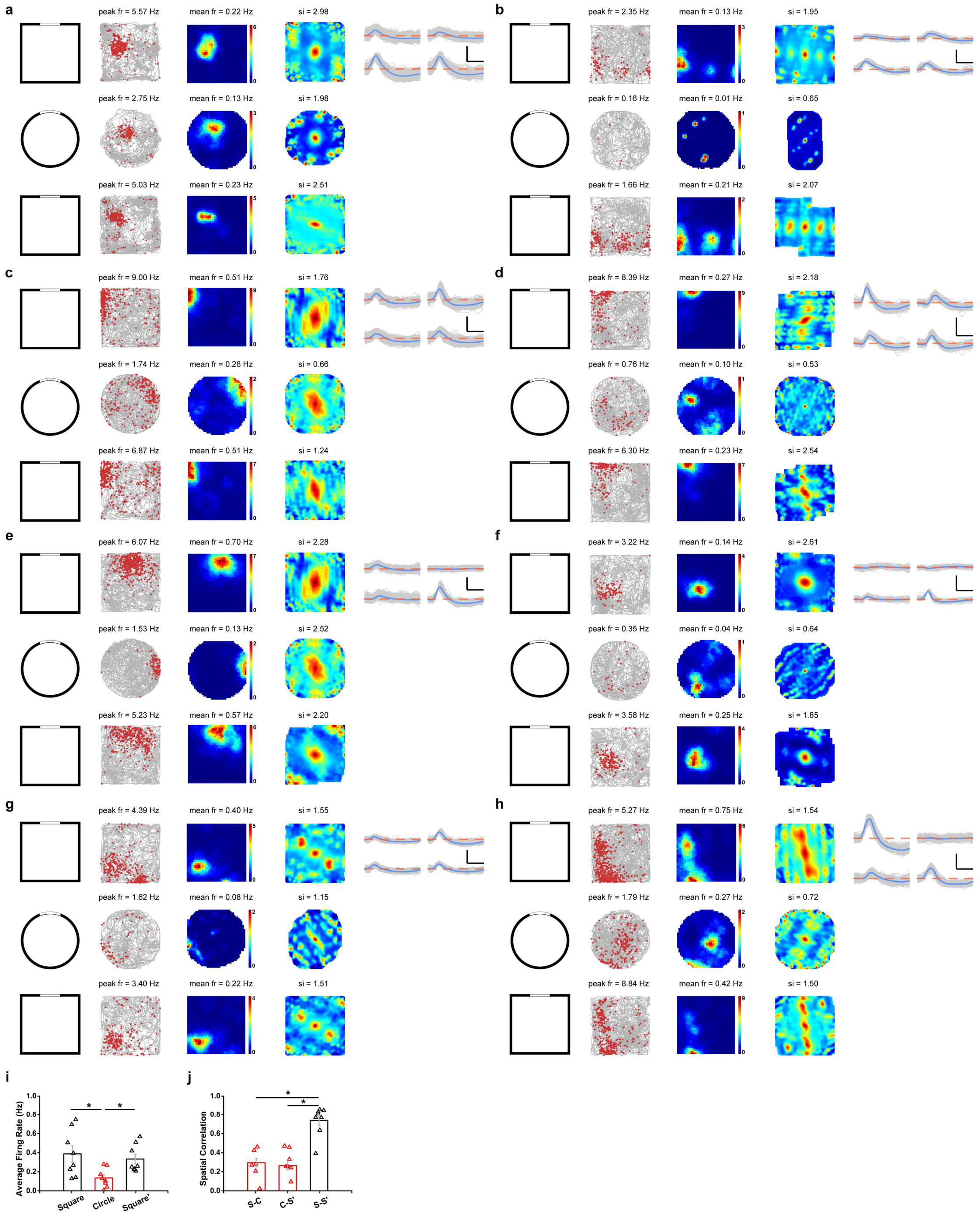

Supplementary information, Fig. S12. Remapping of somatosensory place cells in different environments.

a-h Spatial responses of six representative somatosensory place cells in different boxes in a constant location. The experimental diagram (left column); trajectory (grey line) with superimposed spike locations (red dots) (middle left column); spatial firing rate maps (middle right column) and autocorrelation diagrams (right column). Firing rate is

color-coded with blue indicating minimum firing rate and red indicating maximum firing rate. The scale of the autocorrelation maps is twice that of the spatial firing rate maps. Peak firing rate (fr), mean firing rate (fr) and spatial information (si) for each recording session are labelled at the top of the panels. Spike waveforms on four electrodes are shown on the right column. The zero microvolt horizontal baseline is drawn with the orange dashed lines for the spike waveforms on all four electrodes. Scale bar, 150 μV, 300 μs.

i, j The comparison of the average firing rate and the spatial correlation between rate maps in running boxes under different geometric shapes.
