# Supplementary material for: A novel somatosensory spatial navigation system outside the hippocampal formation
Source: Cell Res. 2021 Jan 18;31(6):649–63. doi: 10.1038/s41422-020-00448-8 (PMC8169756; doi:10.1038/s41422-020-00448-8)
Supplement: Supplementary file 13 — Figure S13 [file 41422_2020_448_MOESM13_ESM.pdf]

Supplementary information, Fig. S13

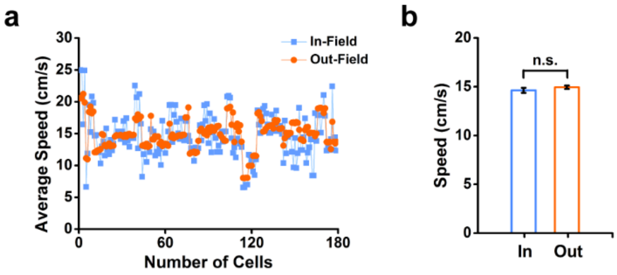

Supplementary information, Fig. S13. Distribution of the average in-field and out-field running speed for place cells in the somatosensory cortex.

a The distribution of the average running speed within and outside the firing fields of all identified place cells in the somatosensory cortex.

b The comparison of the average in-field and out-field running speed. $n = 195$, $P = 0.10$, two-tailed paired t-test, n.s., not significant.
