# Supplementary material for: A novel somatosensory spatial navigation system outside the hippocampal formation
Source: Cell Res. 2021 Jan 18;31(6):649–63. doi: 10.1038/s41422-020-00448-8 (PMC8169756; doi:10.1038/s41422-020-00448-8)
Supplement: Supplementary file 16 — Figure S17 [file 41422_2020_448_MOESM16_ESM.pdf]

## Supplementary information, Fig. S16

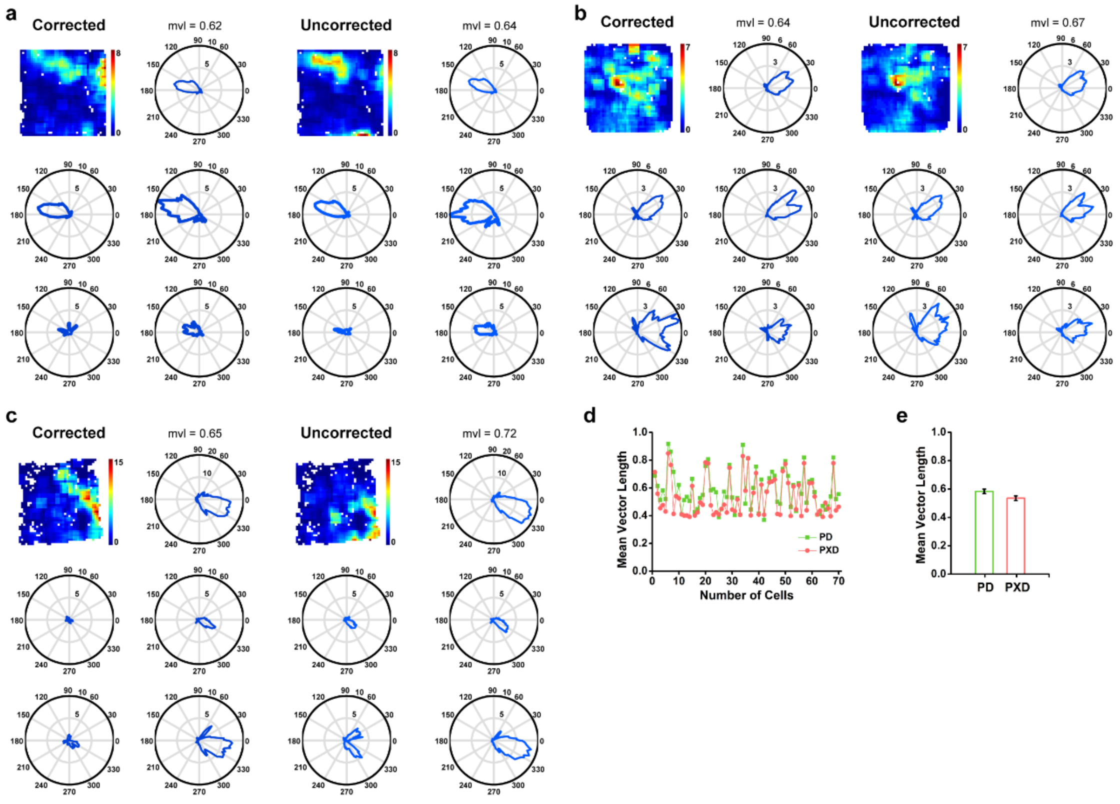


**Supplementary information, Fig. S16. Quantification of head direction selectivity of somatosensory head direction cells using the maximum likelihood factorial model.**

**a-c** Comparison of head directionality of three representative head direction cells from Fig. 2a using a maximum-likelihood approach. Top left two panels show the corrected rate maps and polar plots under the maximum likelihood factorial model. Top right two panels show the uncorrected histograms. Mean vector length (mvl) is indicated at the top right corner of the polar plot panel. The head direction responses in each quadrant of the running box are shown in the four panels below. Somatosensory HD cells preserve the same sharp head direction selectivity in different parts of the running box regardless of possible inhomogeneous sampling of the animal’s locations and orientations.

**d, e** The distribution and the average value of the directional information of head direction cells before (PD) and after (PXD) applying the maximum-likelihood correction algorithm.
