# Supplementary material for: A novel somatosensory spatial navigation system outside the hippocampal formation
Source: Cell Res. 2021 Jan 18;31(6):649–63. doi: 10.1038/s41422-020-00448-8 (PMC8169756; doi:10.1038/s41422-020-00448-8)
Supplement: Supplementary file 17 — Figure S18 [file 41422_2020_448_MOESM17_ESM.pdf]

Supplementary information, Fig. S17

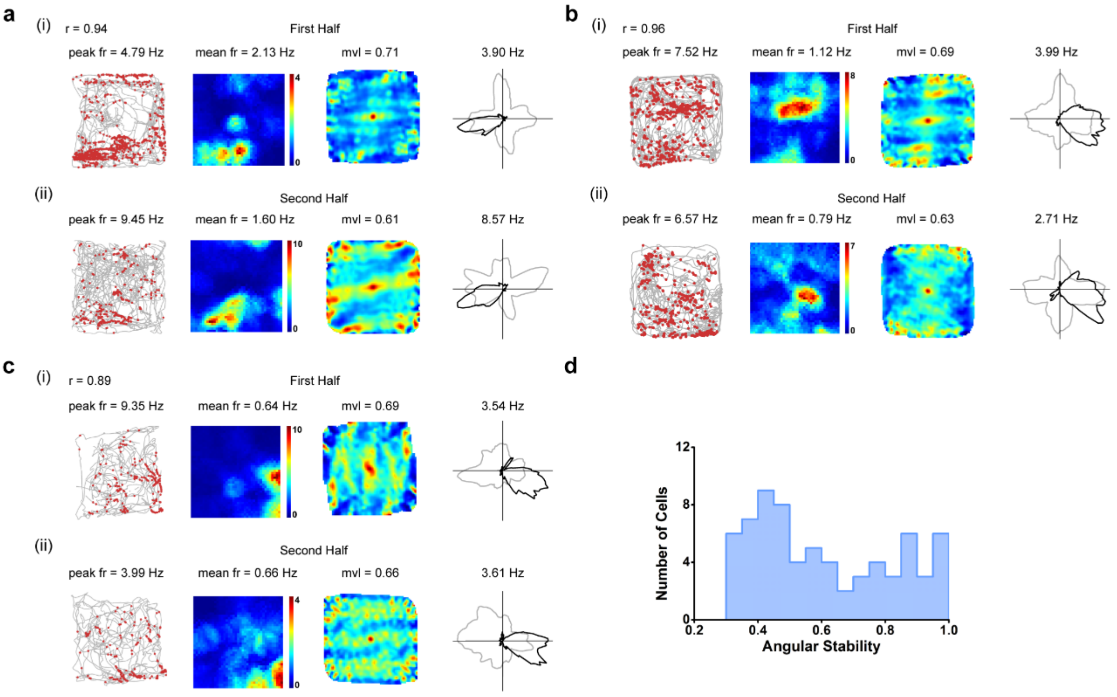

Supplementary information, Fig. S17. Angular stability of somatosensory head direction cells.

a-c Intra-trial angular stability between the first and second halves of three representative head direction cells from Fig. 2a. Trajectory (grey line) with superimposed spike locations (red dots) (left column); spatial firing rate maps (middle left column), autocorrelation diagrams (middle right column) and head direction tuning curves (black) plotted against dwell-time polar plot (grey) (right column) for the first half (**i**) and the second half (**ii**) of the trials. Firing rate is color-coded with blue indicating minimum firing rate and red indicating maximum firing rate. The scale of the autocorrelation maps is twice that of the spatial firing rate maps. Peak firing rate (fr), mean firing rate (fr), mean vector length (mvl) and angular peak rate for each representative head direction cell are labelled at the top of the panels. Correlation coefficients of the distributed firing rate across all directional bins between the first and second halves of individual recording trials are indicated with *r*.

d Population histogram of angular stability of the distributed firing rate across all directional bins between the first and second halves of all identified somatosensory head direction cells.
