# Supplementary material for: A novel somatosensory spatial navigation system outside the hippocampal formation
Source: Cell Res. 2021 Jan 18;31(6):649–63. doi: 10.1038/s41422-020-00448-8 (PMC8169756; doi:10.1038/s41422-020-00448-8)
Supplement: Supplementary file 18 — Figure S16 [file 41422_2020_448_MOESM18_ESM.pdf]

Supplementary information, Fig. S18

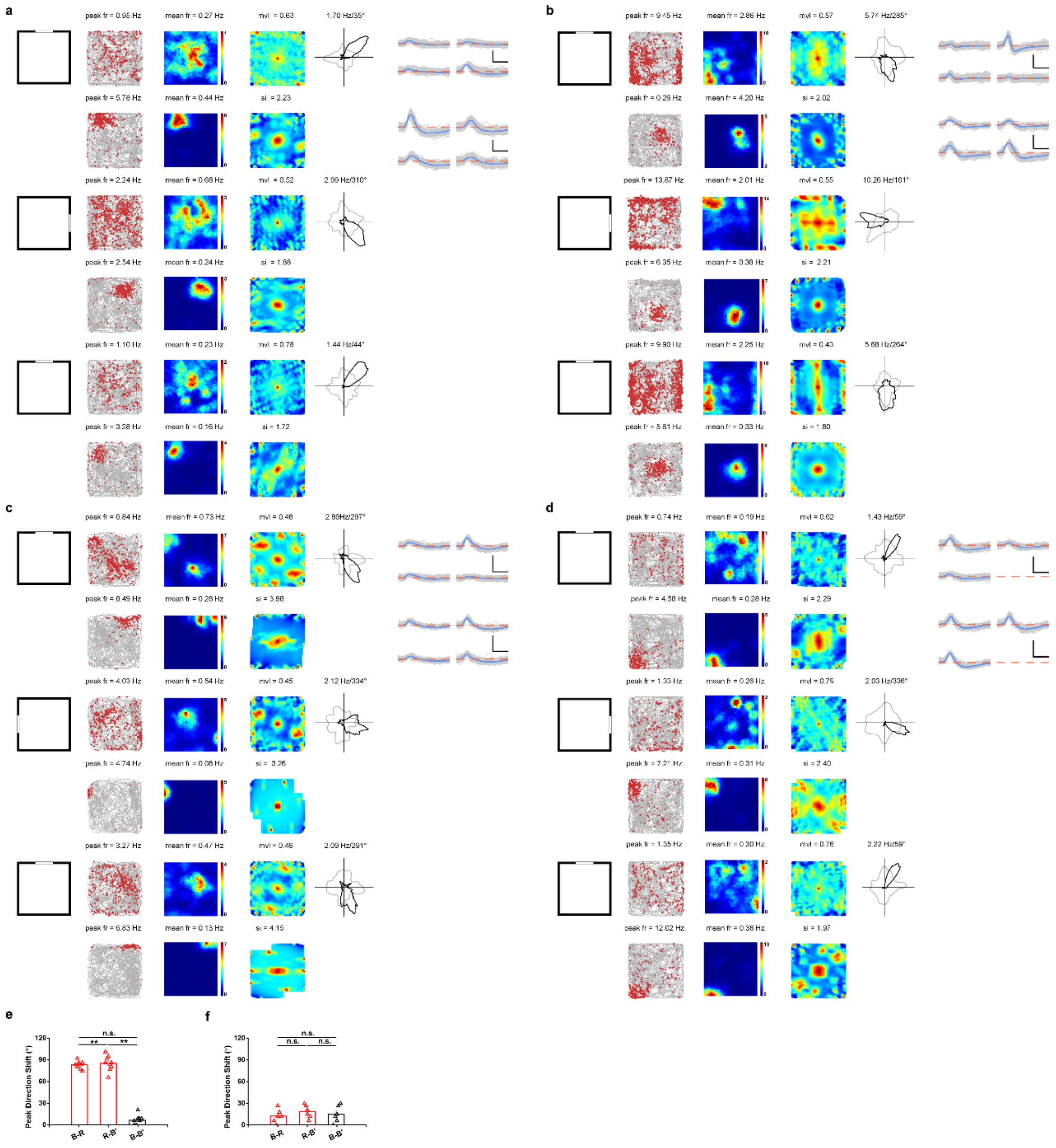

Supplementary information, Fig. S18. Cue-rotation manipulations of somatosensory head directional responses.

a-d Spatial responses of four pairs of co-recorded somatosensory head direction cells and place cells during cue-rotation test. Each panel shows the response of the same S1 head direction cell or place cell during three recording trails under the cue-rotation condition. Top panels, the first standard baseline session (B) before the cue-rotation; middle panels, 90° clockwise or counter-clockwise cue-rotation (R); bottom panels, cue-rotation back to the second baseline condition (B’). The cue card is represented by a white line in each panel. The experimental diagram (left column); trajectory (grey

line) with superimposed spike locations (red dots) (middle left column); spatial firing rate maps (middle column), autocorrelation diagrams (middle right column) and head direction tuning curves (black) plotted against dwell-time polar plot (grey) (right column) for each recording trial. Firing rate is color-coded with blue indicating minimum firing rate and red indicating maximum firing rate. The scale of the autocorrelation maps is twice that of the spatial firing rate maps. Peak firing rate (fr), mean firing rate (fr), mean vector length (mvl) and angular peak rate for each representative head direction cell are labelled at the top of the panels. Spike waveforms on four electrodes are shown on the right column. The zero microvolt horizontal baseline is drawn with the orange dashed lines for the spike waveforms on all four electrodes. Scale bar, 150 µV, 300 µs.

e, f The comparison of the peak direction shift of head direction cells (n = 9) following cue card rotation and head direction cells (n = 6) not following cue card rotation.
