# Supplementary material for: A novel somatosensory spatial navigation system outside the hippocampal formation
Source: Cell Res. 2021 Jan 18;31(6):649–63. doi: 10.1038/s41422-020-00448-8 (PMC8169756; doi:10.1038/s41422-020-00448-8)
Supplement: Supplementary file 19 — Figure S19 [file 41422_2020_448_MOESM19_ESM.pdf]

Supplementary information, Fig. S19

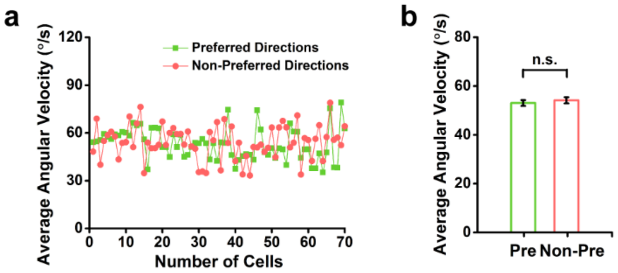

Supplementary information, Fig. S19. Distribution of the average angular velocity in the preferred and non-preferred firing directions for head direction cells in the somatosensory cortex.

a The distribution of the average angular velocity in the "preferred firing directions" and "non-preferred firing directions" for all identified head direction cells in the somatosensory cortex.

b The comparison of the angular velocity in the "preferred firing directions" and "non-preferred firing directions". $n = 70$, $P = 0.47$, two-tailed paired t-test, n.s., not significant.
