# Supplementary material for: A novel somatosensory spatial navigation system outside the hippocampal formation
Source: Cell Res. 2021 Jan 18;31(6):649–63. doi: 10.1038/s41422-020-00448-8 (PMC8169756; doi:10.1038/s41422-020-00448-8)
Supplement: Supplementary file 20 — Figure S20 [file 41422_2020_448_MOESM20_ESM.pdf]

Supplementary information, Fig. S20

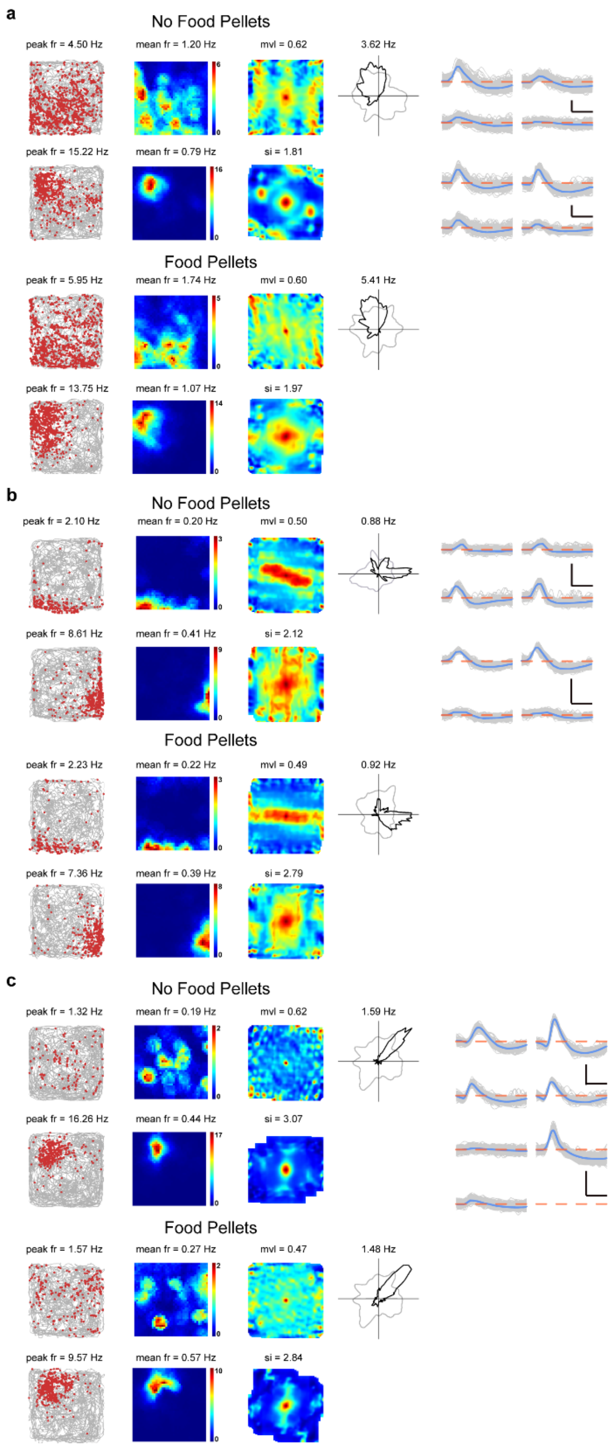

Supplementary information, Fig. S20. Co-recorded somatosensory place cells and head direction cells in the presence or absence of food pellets.

a-c Three pairs of co-recorded S1 head direction cells and place cells with (top two panels) and without (bottom two panels) food pellets. Trajectory (grey line) with superimposed spike locations (red dots) (left column); spatial firing rate maps (middle left column), autocorrelation diagrams (middle right column) and head direction tuning

curves (black) plotted against dwell-time polar plot (grey) (right column). Firing rate is color-coded with blue indicating minimum firing rate and red indicating maximum firing rate. The scale of the autocorrelation maps is twice that of the spatial firing rate maps. Peak firing rate (fr), mean firing rate (fr), mean vector length (mvl) or spatial information (si) and angular peak rate for each representative head direction cell are labelled at the top of the panels. Spike waveforms on four electrodes are shown on the right column. The zero microvolt horizontal baseline is drawn with the orange dashed lines for the spike waveforms on all four electrodes. Scale bar, 150 μV, 300 μs.
