# Supplementary material for: A novel somatosensory spatial navigation system outside the hippocampal formation
Source: Cell Res. 2021 Jan 18;31(6):649–63. doi: 10.1038/s41422-020-00448-8 (PMC8169756; doi:10.1038/s41422-020-00448-8)
Supplement: Supplementary file 21 — Figure S21 [file 41422_2020_448_MOESM21_ESM.pdf]

Supplementary information, Fig. S21

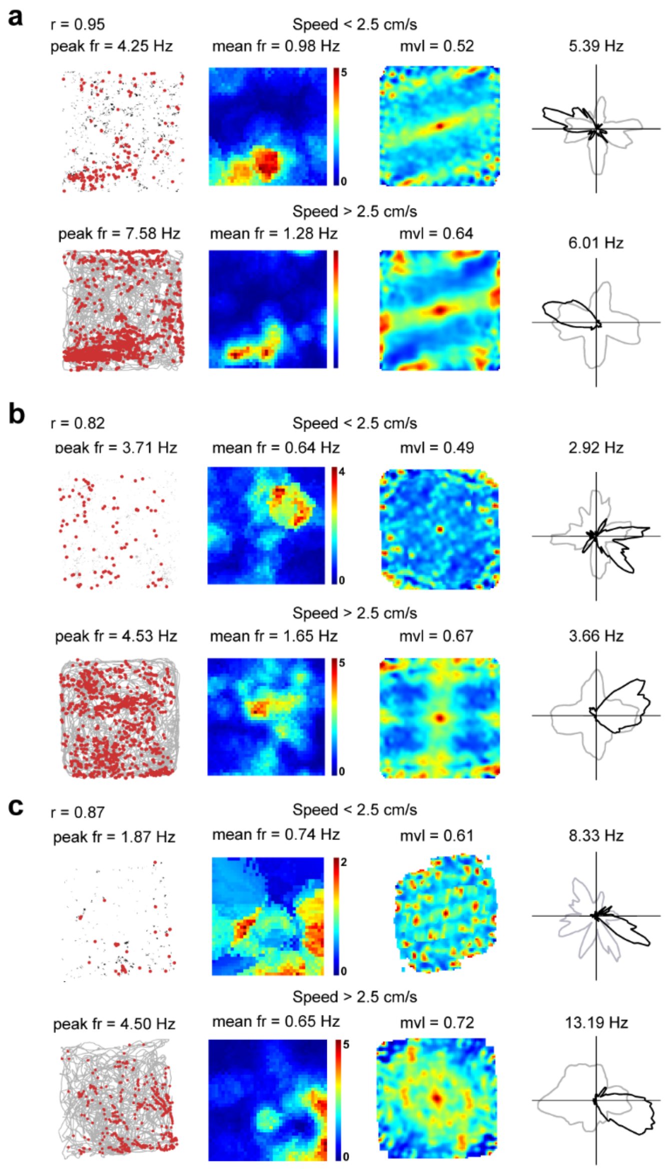

Supplementary information, Fig. S21. Comparison of the spatial response of somatosensory head direction cells between active running and slow immobility.

a-c Comparison of the spatial response of representative head direction cells from Fig. 2a with instantaneous running speeds <2.5 cm/s and instantaneous running speeds > 2.5 cm/s, respectively. Trajectory (grey line) with superimposed spike locations (red dots) (left column); rate maps (middle left column), autocorrelation maps (middle right column) and head direction tuning curves (black) plotted against dwell-time polar plot (grey) (right column) for instantaneous running speeds <2.5 cm/s and instantaneous running speeds > 2.5 cm/s, respectively. Firing rate is color-coded with blue indicating minimum firing rate and red indicating maximum firing rate. The scale of the

autocorrelation maps is twice that of the spatial firing rate maps. Peak firing rate (fr), mean firing rate (fr) and mean vector length (mvl) are labelled at the top of the plots. Correlation coefficients of the distributed firing rate across all directional bins when the instantaneous running speeds <2.5 cm/s and instantaneous running speeds > 2.5 cm/s are indicated with *r* at the top-left corner.
