# Supplementary material for: A novel somatosensory spatial navigation system outside the hippocampal formation
Source: Cell Res. 2021 Jan 18;31(6):649–63. doi: 10.1038/s41422-020-00448-8 (PMC8169756; doi:10.1038/s41422-020-00448-8)
Supplement: Supplementary file 23 — Figure S23 [file 41422_2020_448_MOESM23_ESM.pdf]

Supplementary information, Fig. S23

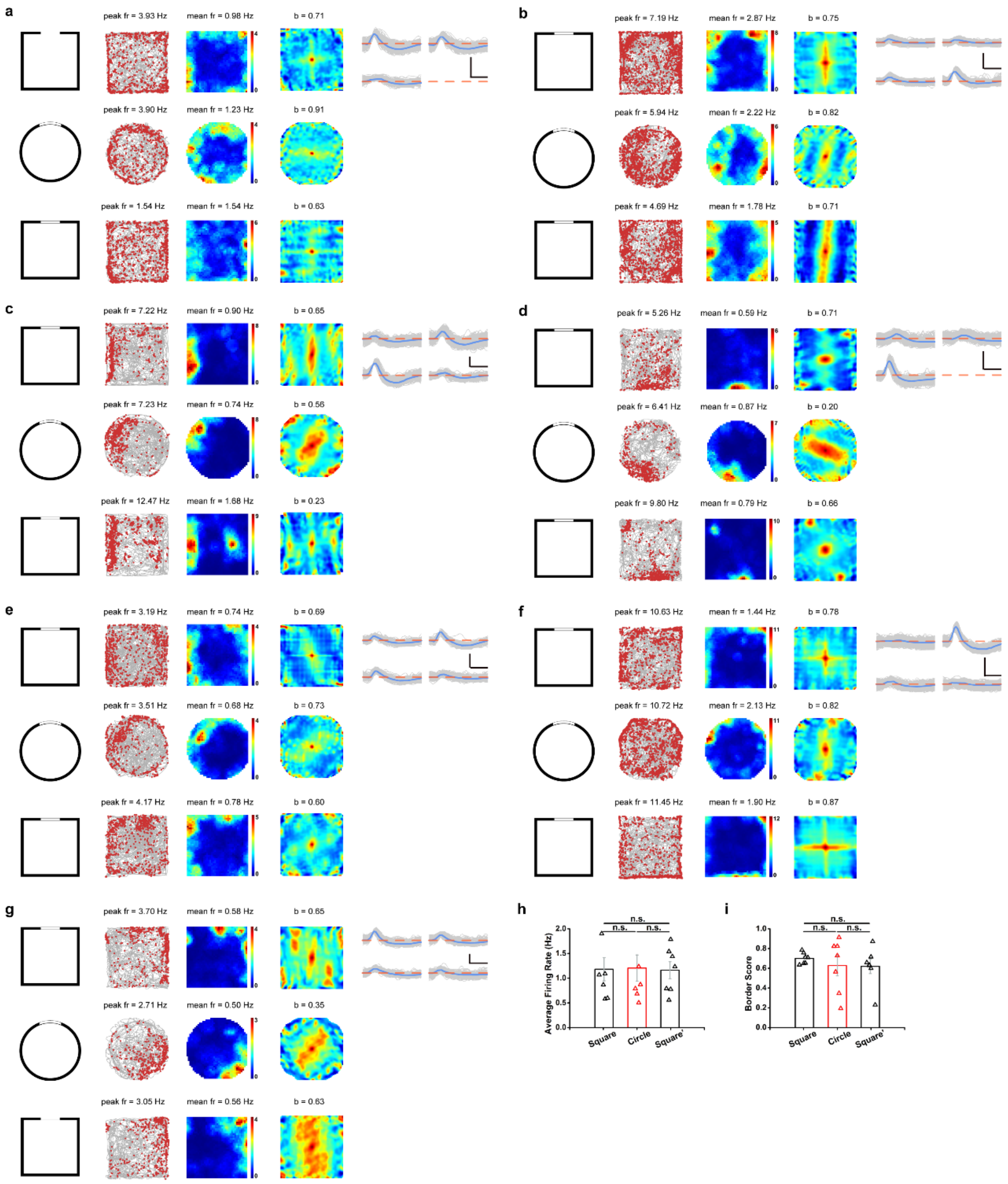

Supplementary information, Fig. S23. Preserved firing across different geometric shapes of somatosensory border cells.

a-g Three representative S1 border cells preserve the firing patterns in the square and cylindrical box. Top panels, original square running box; middle panels, cylindrical box; bottom panels, back to the original square box. The experimental diagram (left column); trajectory (grey line) with superimposed spike locations (red dots) (middle left column); rate maps (middle right column) and autocorrelation diagrams (right column) for each

recording trail. Firing rate is color-coded with blue indicating minimum firing rate and red indicating maximum firing rate. The scale of the autocorrelation maps is twice that of the spatial firing rate maps. Peak firing rate (fr), mean firing rate (fr) and border score (b) for each recording session are labelled at the top of the panels. Spike waveforms on four electrodes are shown on the right column. The zero microvolt horizontal baseline is drawn with the orange dashed lines for the spike waveforms on all four electrodes. Scale bar, 200 μV, 300 μs.

h, i The comparison of the average firing rate and the border score in running boxes with different geometric shapes.
