# Supplementary material for: A novel somatosensory spatial navigation system outside the hippocampal formation
Source: Cell Res. 2021 Jan 18;31(6):649–63. doi: 10.1038/s41422-020-00448-8 (PMC8169756; doi:10.1038/s41422-020-00448-8)
Supplement: Supplementary file 24 — Figure S24 [file 41422_2020_448_MOESM24_ESM.pdf]

Supplementary information, Fig. S24

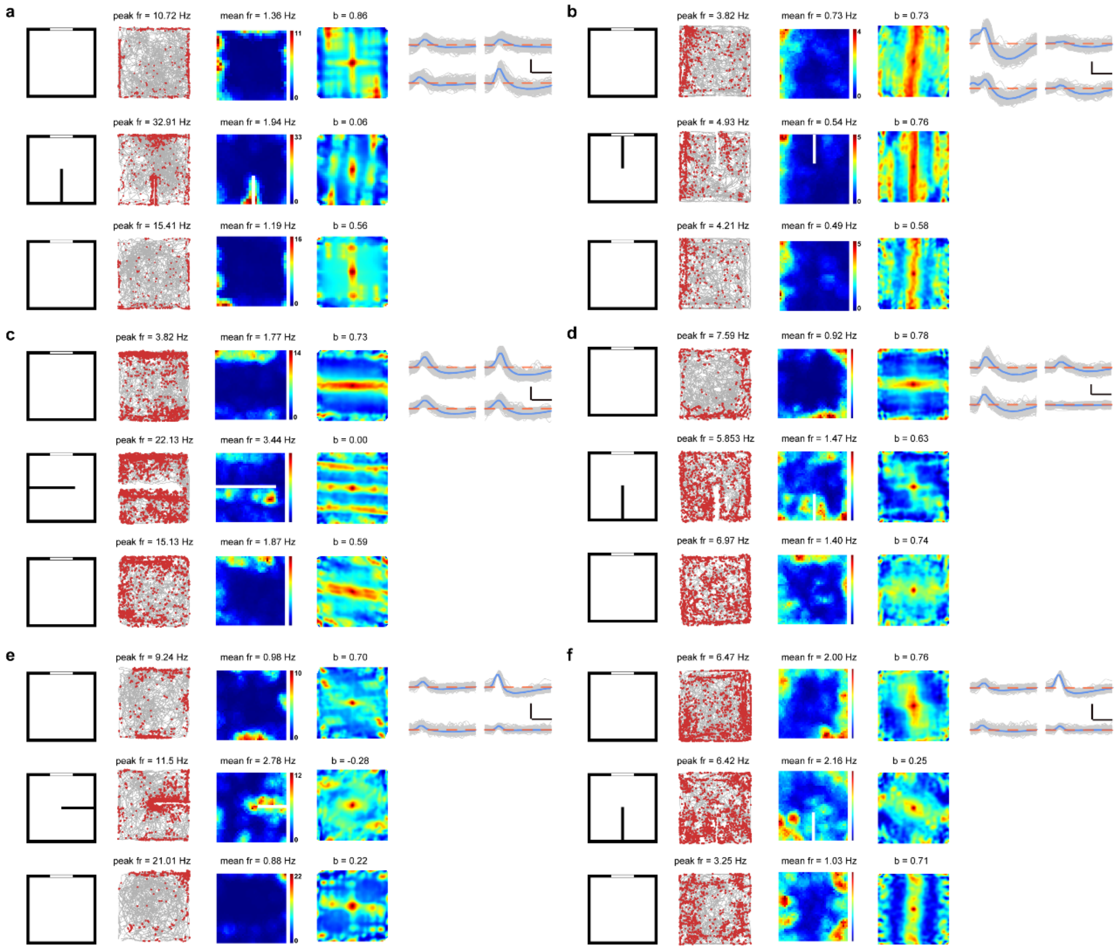

Supplementary information, Fig. S24. Spatial responses to the external inserts of somatosensory border cells.

a-f Six representative somatosensory border cells recorded in the square enclosure with or without one external insert. Top panels, the square enclosure without insert; middle panels, the same square enclosure with an internal insert; bottom panels, the same square enclosure after removing the internal insert. The experimental diagram (left column); trajectory (grey line) with superimposed spike locations (red dots) (middle left column); rate maps (middle right column) and autocorrelation diagrams (right column) for each recording trail. Firing rate is color-coded with blue indicating minimum firing rate and red indicating maximum firing rate. The scale of the autocorrelation maps is twice that of the spatial firing rate maps. Peak firing rate (fr), mean firing rate (fr) and border score (b) for each recording session are labelled at the top of the panels. Spike waveforms on four electrodes are shown on the right column.

The zero microvolt horizontal baseline is drawn with the orange dashed lines for the spike waveforms on all four electrodes. Scale bar, 150 μV, 300 μs.
