# Supplementary material for: A novel somatosensory spatial navigation system outside the hippocampal formation
Source: Cell Res. 2021 Jan 18;31(6):649–63. doi: 10.1038/s41422-020-00448-8 (PMC8169756; doi:10.1038/s41422-020-00448-8)
Supplement: Supplementary file 25 — Figure S25 [file 41422_2020_448_MOESM25_ESM.pdf]

Supplementary information, Fig. S25

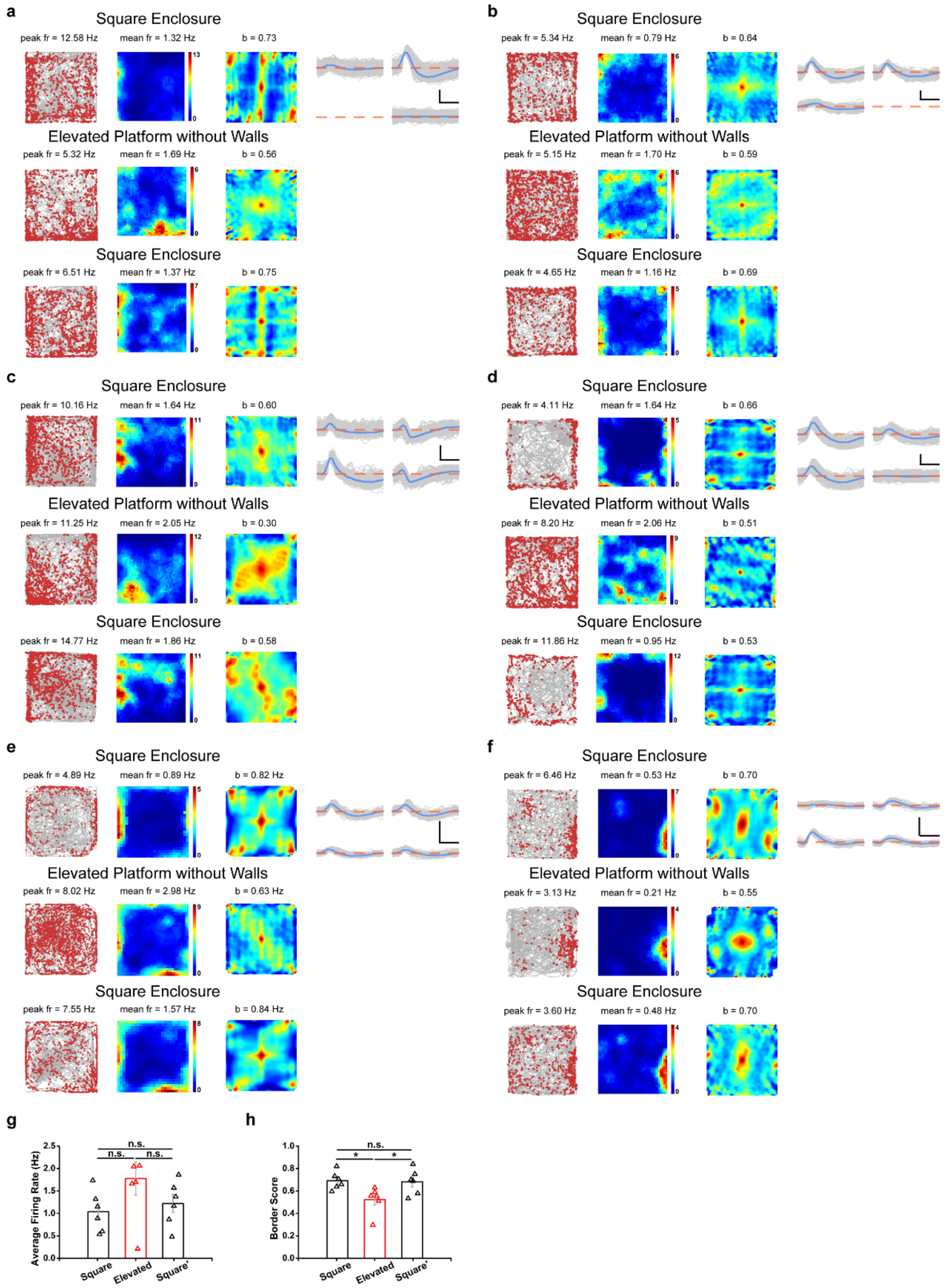

Supplementary information, Fig. S25. Somatosensory border cells recorded from the elevated platform without walls.

a-f Spatial responses of six representative somatosensory border cells recorded in the square box, in the elevated platform without walls and back to the square box.

Trajectory (grey line) with superimposed spike locations (red dots) (left column); heat maps of firing rate (middle column) and autocorrelation maps (right column). Firing rate is color-coded with blue indicating minimum firing rate and red indicating maximum firing rate. The scale of the autocorrelation maps is twice that of the spatial firing rate maps. Peak firing rate (fr), mean firing rate (fr) and border score (b) for each representative border cell are labelled at the top of the panels. Spike waveforms on four electrodes are shown on the right column. The zero microvolt horizontal baseline is drawn with the orange dashed lines for the spike waveforms on all four electrodes. Scale bar, 150 μV, 300 μs.

g, h The comparison of the average firing rate and border score between the square box and the elevated platform.
