# Supplementary material for: A novel somatosensory spatial navigation system outside the hippocampal formation
Source: Cell Res. 2021 Jan 18;31(6):649–63. doi: 10.1038/s41422-020-00448-8 (PMC8169756; doi:10.1038/s41422-020-00448-8)
Supplement: Supplementary file 26 — Figure S26 [file 41422_2020_448_MOESM26_ESM.pdf]

Supplementary information, Fig. S26

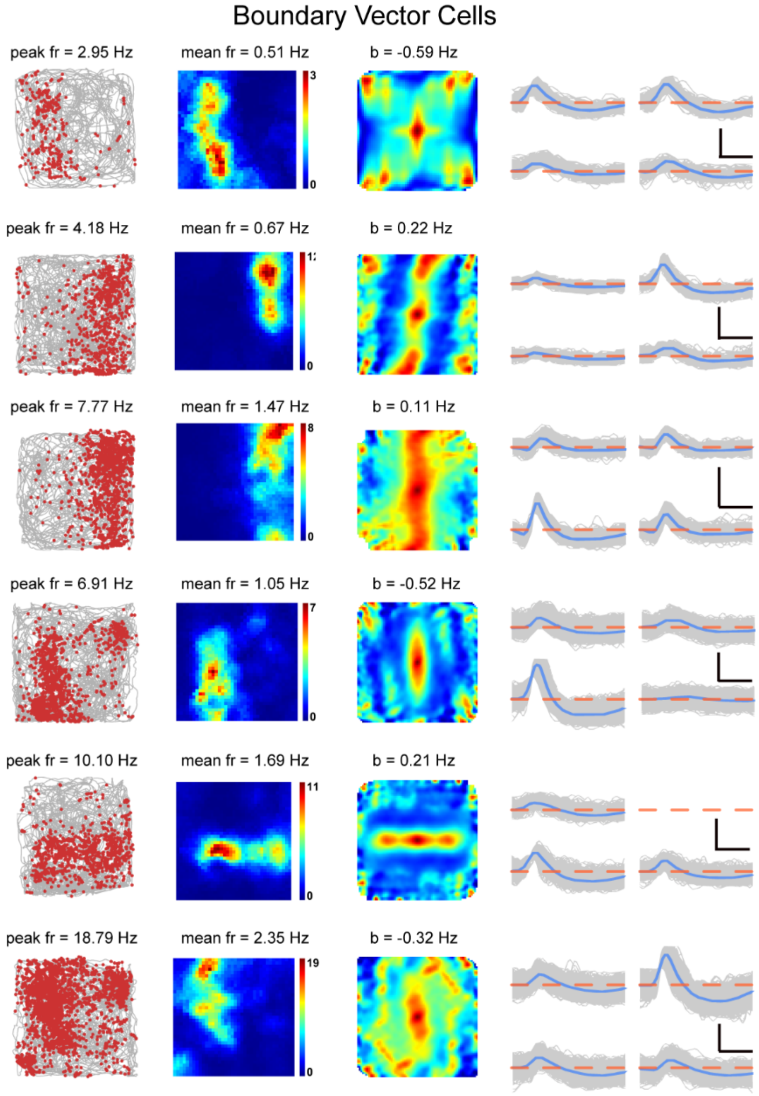

Supplementary information, Fig. S26. Somatosensory boundary vector cells recorded from the somatosensory cortex.

Six examples of somatosensory boundary vector cells. Trajectory (grey line) with superimposed spike locations (red dots) (left column); heat maps of firing rate (middle column) and autocorrelation diagrams (right column). Firing rate is color-coded with blue indicating minimum firing rate and red indicating maximum firing rate. The scale of the autocorrelation maps is twice that of the spatial firing rate maps. Peak firing rate (fr), mean firing rate (fr) and border score (b) for each representative boundary vector cell are labelled at the top of the panels. Spike waveforms on four electrodes are shown on the right column. The zero microvolt horizontal baseline is drawn with the orange dashed lines for the spike waveforms on all four electrodes. Scale bar, 150 µV, 300 µs.
