# Supplementary material for: A novel somatosensory spatial navigation system outside the hippocampal formation
Source: Cell Res. 2021 Jan 18;31(6):649–63. doi: 10.1038/s41422-020-00448-8 (PMC8169756; doi:10.1038/s41422-020-00448-8)
Supplement: Supplementary file 28 — Figure S28 [file 41422_2020_448_MOESM28_ESM.pdf]

Supplementary information, Fig. S28

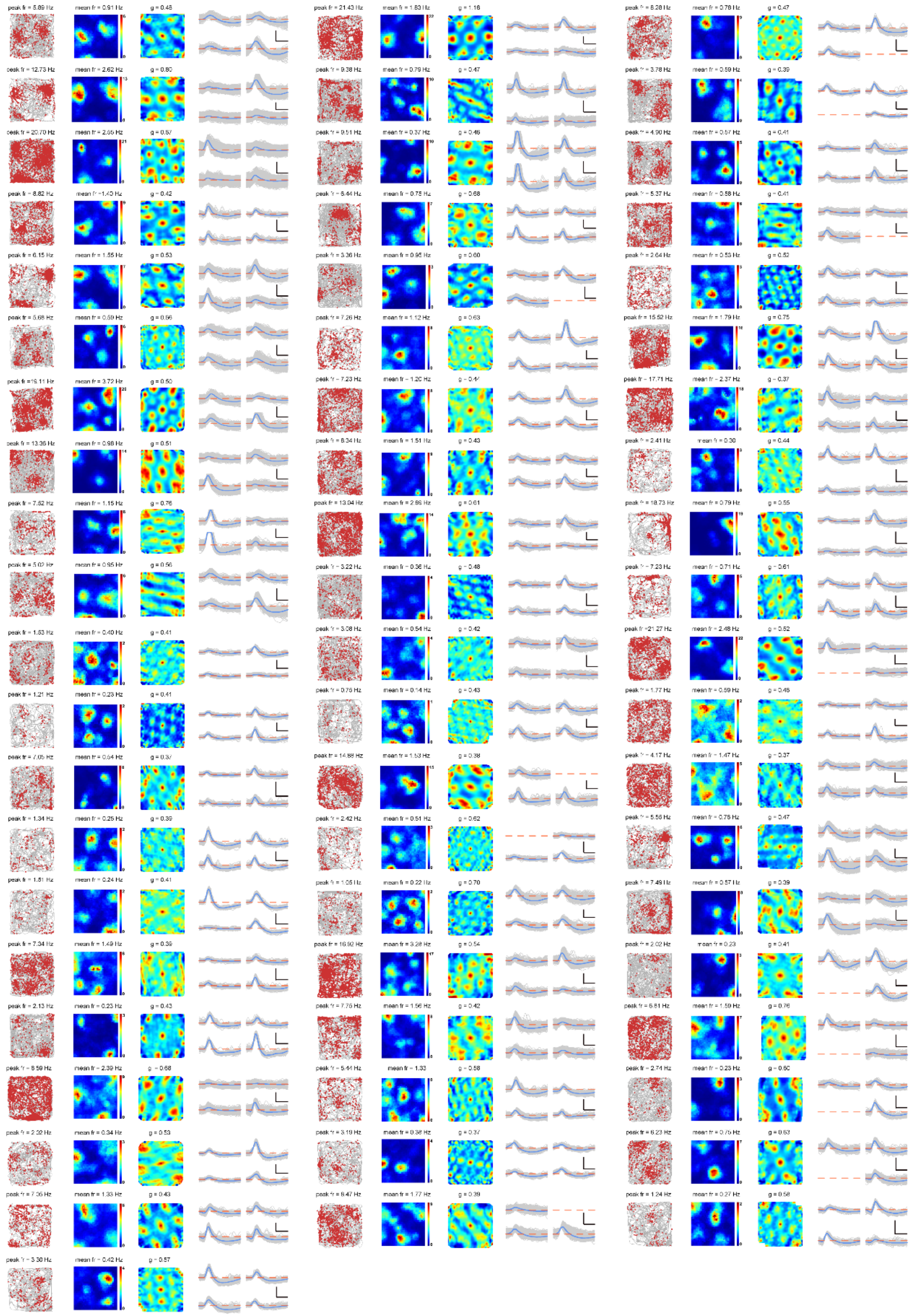

Supplementary information, Fig. S28. All examples of identified grid cells recorded from the somatosensory cortex.

All of identified somatosensory grid cells. Trajectory (grey line) with superimposed

spike locations (red dots) (left column); spatial firing rate maps (middle column) and autocorrelation diagrams (right column). Firing rate is color-coded with dark blue indicating minimal firing rate and dark red indicating maximal firing rate. The scale of the autocorrelation maps is twice that of the spatial firing rate maps. Peak firing rate (fr), mean firing rate (fr) and grid score (g) for each representative head direction cell are labelled at the top of the panels. The directional plots show strong head direction tuning. Spike waveforms on four electrodes are shown on the right column. The zero microvolt horizontal baseline is drawn with the orange dashed lines for the spike waveforms on all four electrodes. Scale bar, 150 μV, 300 μs.
