# Supplementary material for: A novel somatosensory spatial navigation system outside the hippocampal formation
Source: Cell Res. 2021 Jan 18;31(6):649–63. doi: 10.1038/s41422-020-00448-8 (PMC8169756; doi:10.1038/s41422-020-00448-8)
Supplement: Supplementary file 29 — Figure S29 [file 41422_2020_448_MOESM29_ESM.pdf]

Supplementary information, Fig. S29

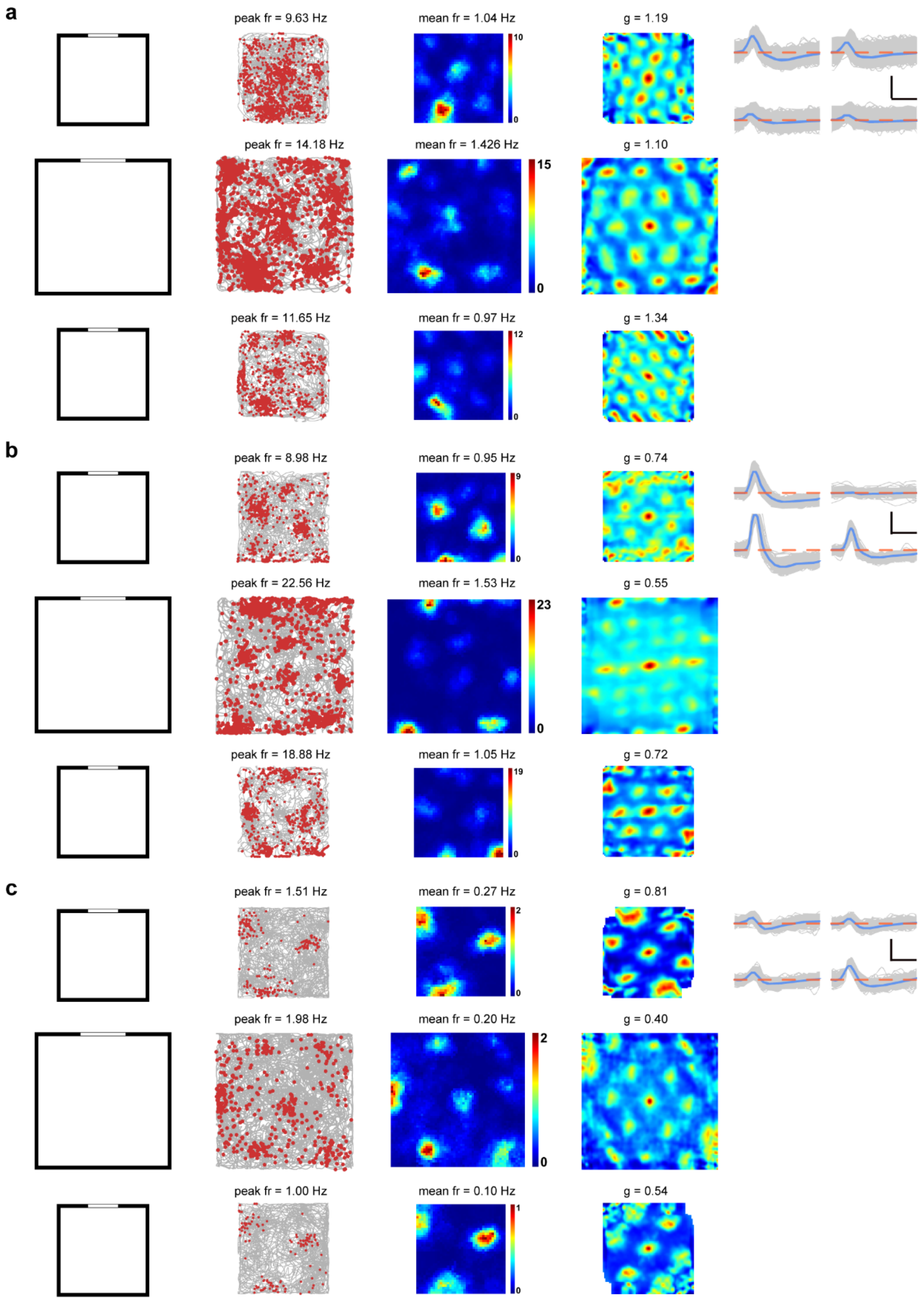

Supplementary information, Fig. S29. Somatosensory grid cells recorded in the larger environment.

a-c Three examples of somatosensory grid cells recorded in 1 m x 1m, 1.5 m x 1.5 m

and 1 m x 1m square box. The experimental diagram (left column); trajectory (grey line) with superimposed spike locations (red dots) (middle left column); spatial firing rate maps (middle right column) and autocorrelation diagrams (right column). Firing rate is color-coded with dark blue indicating minimal firing rate and dark red indicating maximal firing rate. The scale of the autocorrelation maps is twice that of the spatial firing rate maps. Peak firing rate (fr), mean firing rate (fr) and grid score (g) for each representative head direction cell are labelled at the top of the panels. Spike waveforms on four electrodes are shown on the right column. The zero microvolt horizontal baseline is drawn with the orange dashed lines for the spike waveforms on all four electrodes. Scale bar, 150 μV, 300 μs.
