# Supplementary material for: A novel somatosensory spatial navigation system outside the hippocampal formation
Source: Cell Res. 2021 Jan 18;31(6):649–63. doi: 10.1038/s41422-020-00448-8 (PMC8169756; doi:10.1038/s41422-020-00448-8)
Supplement: Supplementary file 30 — Figure S30 [file 41422_2020_448_MOESM30_ESM.pdf]

Supplementary information, Fig. S30

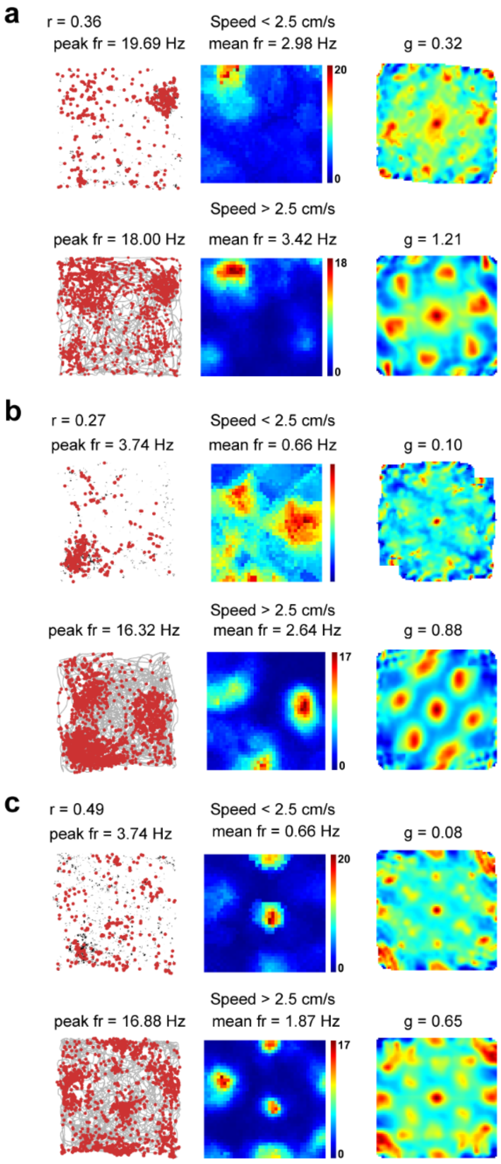

Supplementary information, Fig. S30. Comparison of the spatial response of somatosensory grid cells between active running and slow immobility.

a-c Comparison of the spatial response of representative grid cells from Fig. 4a with instantaneous running speeds <2.5 cm/s and instantaneous running speeds > 2.5 cm/s, respectively. Trajectory (grey line) with superimposed spike locations (red dots) (left column); rate maps (middle column) and autocorrelation maps (right column) for instantaneous running speeds <2.5 cm/s and instantaneous running speeds > 2.5 cm/s, respectively. Firing rate is color-coded with blue indicating minimum firing rate and red indicating maximum firing rate. The scale of the autocorrelation maps is twice that

of the spatial firing rate maps. Peak firing rate (fr), mean firing rate (fr) and grid score (b) are labelled at the top of the plots. Pearson's correlation coefficients between two firing rate maps when the instantaneous running speeds <2.5 cm/s and instantaneous running speeds > 2.5 cm/s are indicated with *r* at the top-left corner.
