# Supplementary material for: A novel somatosensory spatial navigation system outside the hippocampal formation
Source: Cell Res. 2021 Jan 18;31(6):649–63. doi: 10.1038/s41422-020-00448-8 (PMC8169756; doi:10.1038/s41422-020-00448-8)
Supplement: Supplementary file 32 — Figure S32 [file 41422_2020_448_MOESM32_ESM.pdf]

Supplementary information, Fig. S32

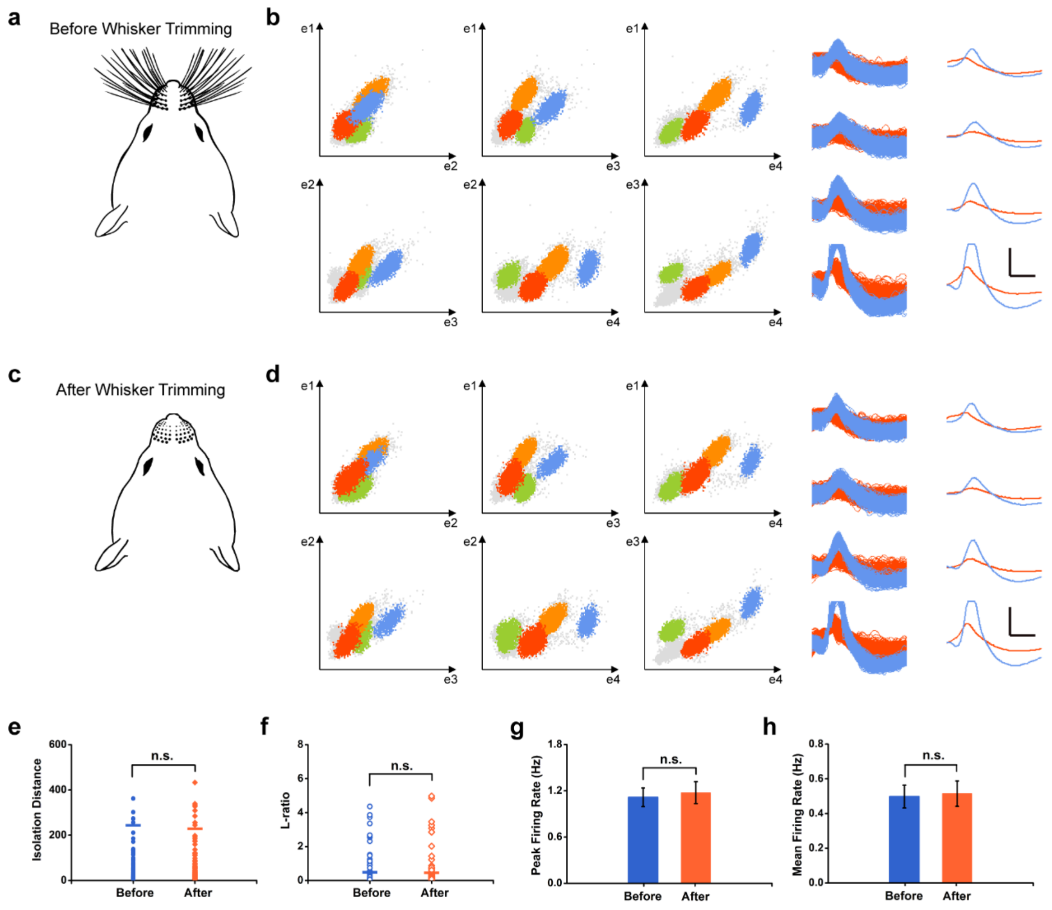

Supplementary information, Fig. S32. Preserved firing properties of the isolated somatosensory units before and after whisker trimming.

a, c The diagram showing the rat before (**a**) and after (**c**) whisker trimming.

b, d Scatterplots show preserved cluster diagrams before (**b**) and after (**d**) whisker trimming of the same tetrode from the same recording rat. Each individual dot represents a single recorded spike. Waveforms from two separated blue and red clusters in the scatterplots are shown for four electrodes from the same tetrode. The waveforms remain unchanged after whisker trimming. Scale bar, 150 μV, 300 μs.

e The comparison of isolation distance for identified S1 units right before and after whisker trimming.

f Same as **e** for the L-ratio.

g Same as in (**f**) for the mean firing rate.

h Same as in (**g**) for the peak firing rate. n = 78, two-tailed paired t-test, n.s., not significant.
