# Supplementary material for: A novel somatosensory spatial navigation system outside the hippocampal formation
Source: Cell Res. 2021 Jan 18;31(6):649–63. doi: 10.1038/s41422-020-00448-8 (PMC8169756; doi:10.1038/s41422-020-00448-8)
Supplement: Supplementary file 33 — Figure S33 [file 41422_2020_448_MOESM33_ESM.pdf]

Supplementary information, Fig. S33

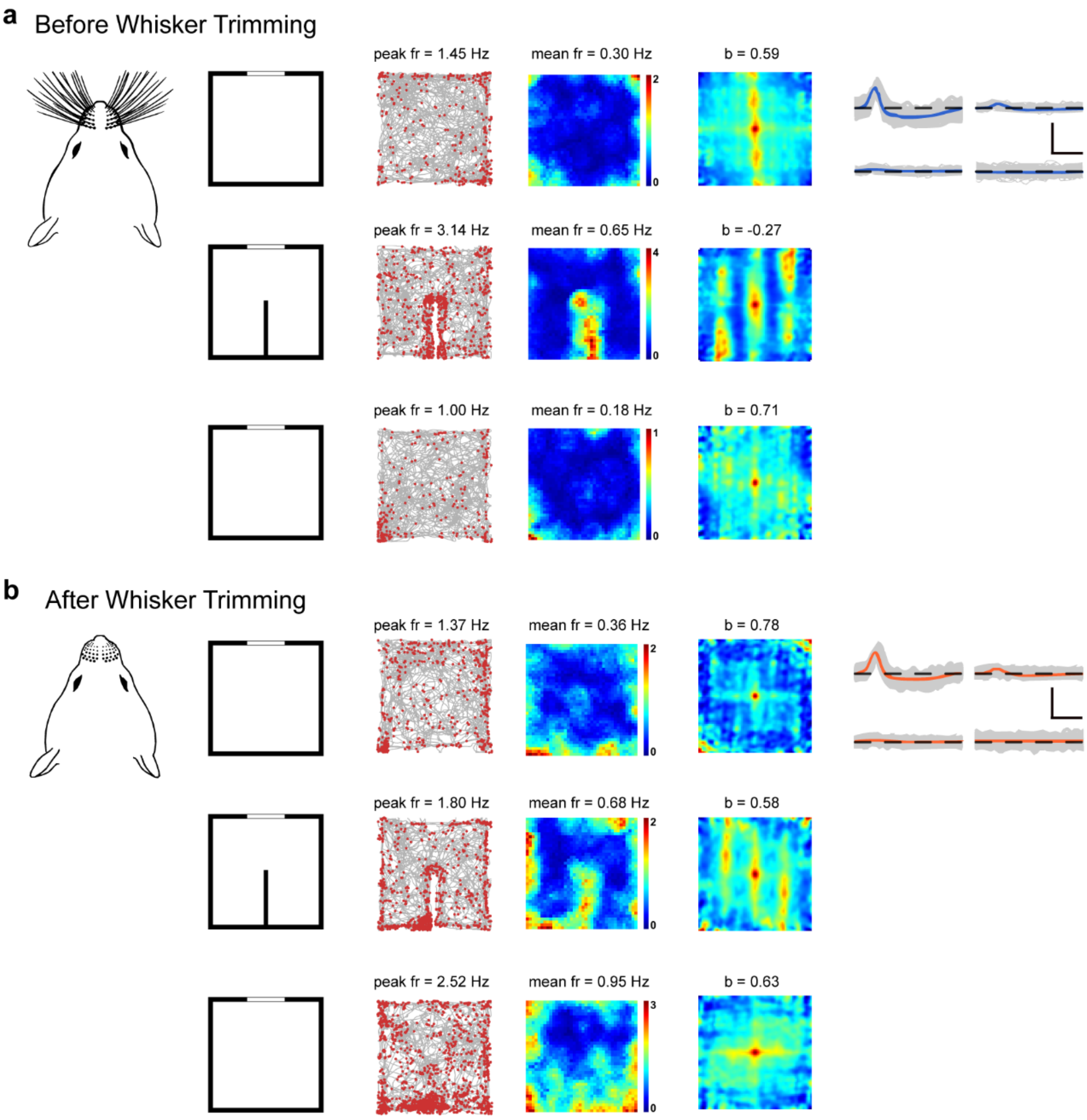

Supplementary information, Fig. S33. Preserved spatial firing properties of the same somatosensory border cell before and after whisker trimming.

a, b The diagram showing the rat before (**a**) and after (**b**) whisker trimming. A representative somatosensory border cell preserves the firing patterns in the square box with (top and bottom panels) and without (middle panels) the internal insert before and after whisker trimming. The experimental diagram (left column); trajectory (grey line) with superimposed spike locations (red dots) (middle left column); rate maps (middle right column) and autocorrelation maps (right column) for each recording trial from the same S1 border cell. Firing rate is color-coded with blue indicating minimum firing rate and red indicating maximum firing rate. The scale of the autocorrelation maps is twice

that of the spatial firing rate maps. Peak firing rate (fr), mean firing rate (fr) and border score (b) for each recording session are labelled at the top of the panels. Spike waveforms on four electrodes are shown on the right column. The zero microvolt horizontal baseline is drawn with the black dashed lines for the spike waveforms on all four electrodes. Scale bar, 100 μV, 300 μs.
