# Supplementary material for: A novel somatosensory spatial navigation system outside the hippocampal formation
Source: Cell Res. 2021 Jan 18;31(6):649–63. doi: 10.1038/s41422-020-00448-8 (PMC8169756; doi:10.1038/s41422-020-00448-8)
Supplement: Supplementary file 34 — Table S1 [file 41422_2020_448_MOESM34_ESM.pdf]

Supplementary information, Table S1

	Somatosensory Spatial Cell Types				
**Rat Number**	**Place Cells**	**Head Direction Cells**	**Border Cells**	**Grid Cells**	**Total Cells**
1 (S1HL)	22	13	6	12	182
2 (S1HL)	27	14	14	13	312
3 (S1FL)	19	7	8	10	353
4 (S1HL)	20	4	12	9	275
5 (S1Sh)	20	5	8	12	263
6 (S1HL)	35	8	16	7	197
7 (S1HL)	36	8	12	5	301
8 (S1HL)	16	11	10	4	142
**Total**	195	70	86	72	2025

Supplementary information, Table. S1. Summary of somatosensory spatial cell types recorded in each rat.
